# Discovery of lead quinone cathode materials for Li-ion batteries†

**DOI:** 10.1039/d2dd00112h

**Published:** 2023-05-30

**Authors:** Xuan Zhou, Abhishek Khetan, Jie Zheng, Mark Huijben, René A. J. Janssen, Süleyman Er

**Affiliations:** a DIFFER – Dutch Institute for Fundamental Energy Research De Zaale 20 5612 AJ Eindhoven the Netherlands s.er@differ.nl; b Department of Applied Physics, Eindhoven University of Technology Eindhoven 5600 MB the Netherlands; c Molecular Materials and Nanosystems, Institute for Complex Molecular System, Eindhoven University of Technology Eindhoven 5600 MB the Netherlands; d Multiscale Modeling of Heterogeneous Catalysis in Energy Systems, RWTH Aachen University Aachen 52062 Germany; e MESA+ Institute for Nanotechnology, University of Twente Enschede 7500 AE the Netherlands

## Abstract

Organic cathode materials are attractive candidates for the development of high-performance Li-ion batteries (LIBs). The chemical space of candidate molecules is too vast to be explored solely by experiments; however, it can be systematically explored by a high-throughput computational search that incorporates a spectrum of screening techniques. Here, we present a time- and resource-efficient computational scheme that incorporates machine learning and semi-empirical quantum mechanical methods to study the chemical space of approximately 200 000 quinone-based molecules for use as cathode materials in LIBs. By performing an automated search on a commercial vendor database, computing battery-relevant properties such as redox potential, gravimetric charge capacity, gravimetric energy density, and synthetic complexity score, and evaluating the structural integrity upon the lithiation process, a total of 349 molecules were identified as potentially high-performing cathode materials for LIBs. The chemical space of the screened candidates was visualized using dimensionality reduction methods with the aim of further downselecting the best candidates for experimental validation. One such directly purchasable candidate, 1,4,9,10-anthracenetetraone, was analyzed through cyclic voltammetry experiments. The measured redox potentials of the two lithiation steps, 
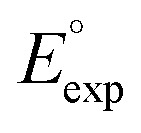
, of 3.3 and 2.4 V, were in good agreement with the predicted redox potentials, 
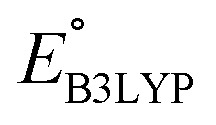
, of 3.2 and 2.3 V *vs.* Li/Li^+^, respectively. Lastly, to lay out the principles for rational design of quinone-based cathode materials beyond the current work, we constructed and discussed the quantitative structure property relationships of quinones based on the data generated from the calculations.

## Abstract 2

The search for quinone-based lithium-ion battery cathode materials within a vast chemical space.

## Graphical abstract

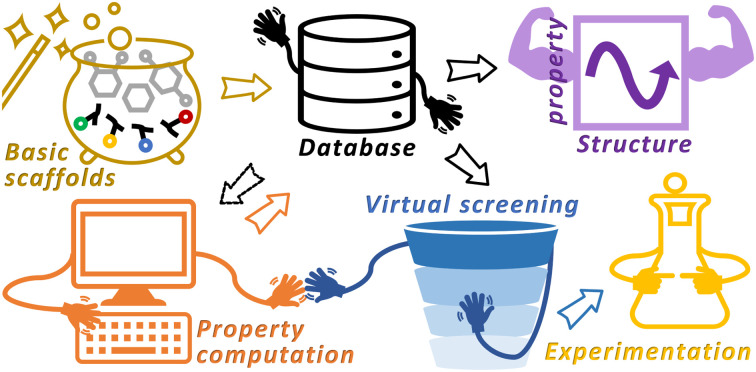

## 1. Introduction

Redox-active organic materials, including carbonyls,^1^ conducting polymers,^2^ organosulfur compounds,^3^ organic radicals,^4^ and imine compounds^5^ are emerging as promising electrode alternatives to their inorganic counterparts for Li-ion batteries (LIBs) owing to their abundant source elements, sustainable synthesizability, and structural and electrochemical property tunability. Among the variety of organic motifs, conjugated carbonyls are one of the earliest investigated energy storage materials for battery applications, whose history dates back to the 1970s.^6^ To date, carbonyl-containing organics, such as quinones,^7^ aromatic imides,^8^ anhydrides,^9^ and ketones,^10^ have been explored as electrode materials for LIBs.

Quinone-based small molecules are particularly attractive in the research community as they offer high theoretical specific capacity and promising redox stability and can be sourced from biomass.^7,11^ However, in comparison to state-of-the-art inorganic cathode materials, small quinones have not yet been found to be sufficiently viable in terms of actual redox potentials and cycling stability.^12–14^ Improvements in the electrochemical performance of quinone-based cathode materials are possible by fine-tuning their molecular structure through different approaches, including functionalization with *R*-groups,^15^ fusing aromatic^16^ or heteroaromatic rings^17^ together, and incorporation of additional redox-active carbonyl groups.^18^

Going beyond the Edisonian experimental approach of trial-and-error, high-throughput computational screening (HTCS) has emerged as a powerful approach for accelerating the discovery of high-performing material candidates. Owing to the ever-increasing computational power and the new analysis tools that are based on robust theoretical approaches, HTCS has become an efficient means for the exploration of large ‘virtual chemical spaces’.^19–24^ However, previous computational studies on quinone-based LIB cathode materials have been restricted to a modest number of molecules which were derived from a limited range of often intuitively chosen quinone core structures, including cyclohexa-2,5-diene-1,4-dione (BQ),^25–27^ naphthalene-1,4-dione (NQ),^25,28,29^ anthracene-9,10-dione (AQ),^29–34^ and phenanthrene-9,10-dione (PQ).^17,29,34^ These investigations of chemical spaces, although around few quinone core structures, have offered useful insights on the effectiveness of various strategies, such as functionalization and ring-fusion. However, the effect of these strategies, especially in terms of often competing performance requirements, has not yet been sufficiently well explored for a large range of quinones that span across several functional groups, ring numbers and the number of redox-active carbonyl groups that can interact with Li.

**Fig. 1:**
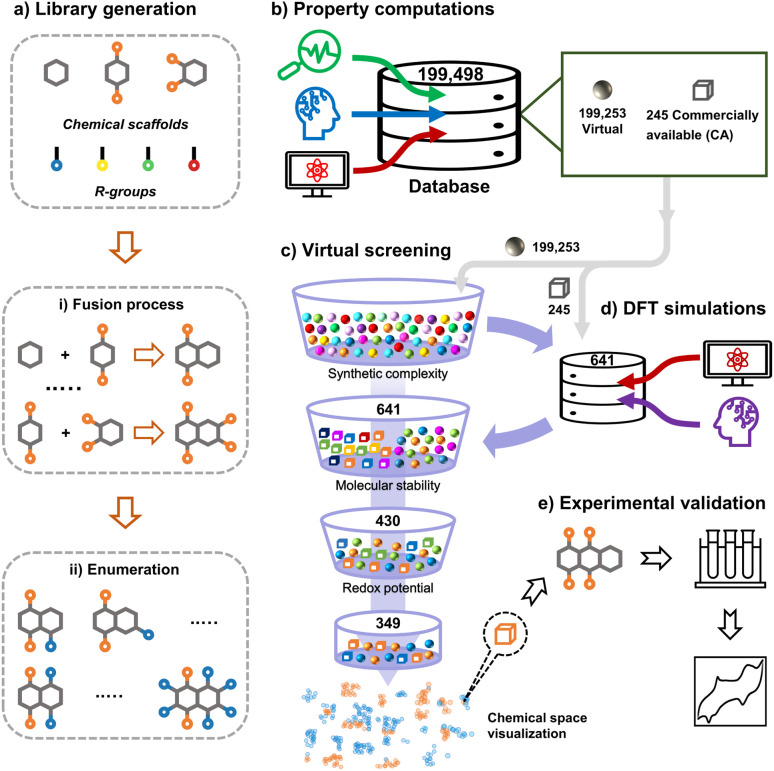
An overview of the data-driven computational and experimental workflow that has been applied for the exploration of the quinone-based LIB cathode materials in the current study.

In this work, we tapped into a large chemical space of systematically enumerated quinones by means of HTCS with two principal goals: (1) finding the top-performing candidates which can be validated experimentally and (2) deciphering the trends in battery performance-related properties induced by pondered molecular engineering. The structure-property relationships emerging from the second step can be used as guiding design principles for future discovery efforts. The workflow of chemical space exploration of organic molecules conceptualized and applied in this work is shown in Fig. 1. The HTCS approach has been carried out in five major steps:

• *Library generation*: a virtual library consisting of 199 498 unique quinone molecules was designed and developed by employing three elementary molecular building-blocks and four chemically different *R*-groups.

• *Property computations*: an automated search for the commercial availability of the virtual library molecules in the ZINC database^35^ was performed, in which matching records for a total of 245 compounds were found. In addition, the simplified molecular input line entry system (SMILES) representations of all the virtual library molecules were used as inputs for a machine learning (ML) model that predicts the synthetic complexity score (SCScore) of the molecules. Next, we employed semi-empirical quantum mechanics (SEQM) calculations to optimize the geometries of molecules and to calculate their redox potentials *via* a linear regression model. Furthermore, the theoretical gravimetric charge capacity (*Q*) and energy density (*W*) of the virtual molecules were calculated.

• *DFT simulations*: a small proportion of the entire library of 199 498 molecules, which were either commercially available or easily synthesizable, was treated with density functional theory (DFT) calculations. Accordingly, 641 molecules were further optimized and their properties were recalculated at a higher level of accuracy. In addition, to determine the stability of molecules, we used a structural alignment algorithm and analyzed the deviations between DFT-optimized structures of the pre- and post-lithiated molecules.

• *Virtual screening*: out of the 641 candidates that have been considered in DFT-calculations, 430 molecules have satisfied the molecular stability criterion enforced in the current work. Moreover, 349 of these 430 molecules have met the prescribed criterion for redox potential. Lastly, we employed the Uniform Manifold Approximation and Projection (UMAP) dimension reduction technique in combination with the tailored similarity method in order to obtain a non-linear dimensional reduction of the top-candidate molecular chemical space.

• *Experimental validation*: for real-lab validation, we performed electrochemical measurements on a readily purchasable molecule from the list of top-compounds that had been identified *via* the virtual screening.

In addition to providing a list of top-grade quinone-based candidate molecules for LIBs, we also included here a structure–property analysis of the entire virtual library molecules.

## 2. Methodology

### 2.1. Virtual library generation and vendor search

Fig. 1 shows the material discovery workflow implemented in this work. The first step (Fig. 1a) was the generation of a virtual library by enumerating all possible quinone molecules within some well-defined compositional constraints. For achieving this, the core quinone structures (CQSs) of the virtual library were first created systematically by combining three elementary building blocks: 1,4-benzoquinone (carbonyl groups at the *para* position), 1,2-benzoquinone (carbonyl groups at the *ortho* position) and benzene. The usage of 1,4- and 1,2-benzoquinones as building blocks for the core structures theoretically promotes a 2e^−^–2Li^+^ reaction, thus enabling ring aromatization during the lithiation process.^12^ The building blocks were then fused to exhaustively generate all possible CQSs that had a maximum of four six-membered rings. This constraint was implemented based on the expectation that the single-molecule approximation for prediction of cathode properties will likely become more erroneous with increasing number of rings due to increasing π-stacking interactions.^36^

In addition to the size constraint, the CQSs contained either two or four C=O groups; in other words, a maximum of two quinone building block molecules per CQS were allowed. Applying these two structural constraints, 170 unique CQSs were generated, which can be classified into five main groups based on the number and position of carbonyl groups. The molecules that consist of one 1,4-benzoquinone ([P]), one 1,2-benzoquinone ([O]), two 1,4-benzoquinone ([P + P]), two 1,2-benzoquinone ([O + O]), and one 1,4-benzoquinone and one 1,2-benzoquinone ([P + O]), are shown in Table 1. Accordingly, the molecular skeletons of [P] and [O] can undergo one pair of lithiation reactions leading to aromatization, whereas [P + P], [O + O], and [P + O] can undergo two pairs of lithiation reactions.

**Table 1:** (Top) Classification of lithiation sites that are found on the CQSs from the virtual library. (Below) A summary of the virtual screening library that has been created, containing all the functionalized molecules obtained through the enumeration of CQSs using four distinct *R*-groups

Type of carbonyl groups			
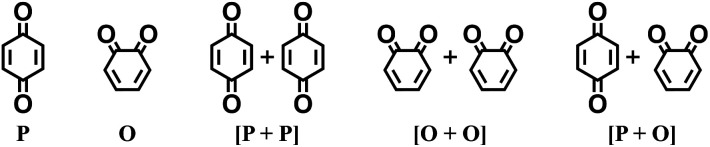			
Number of rings	Number of CQSs	Maximum number of functionalized positions	Number of functionalized molecules
1	2	4	62
2	9	6	793
3	32	8	11 696
4	127	10	186 947
Total	170	—	199 498

Finally, the CQSs were functionalized by replacing the peripheral H atoms on the rings with four different *R*-groups: –Cl, –CH_3_, –OCH_3_, and –NH_2_. These electron-withdrawing and electron-donating chemical groups were chosen to enlarge the electrochemical window of the CQSs.^13,37,38^ Each CQS was exhaustively enumerated using a single type of *R*-group without combining different *R*-groups. The range of functionalized positions of *R*-groups varied between zero and the maximum number of H atoms that are present on that specific CQS. The enumeration of the virtual library was performed automatically using the Custom *R*-Group Enumeration tool as implemented in Schrödinger Materials Science Suite (SMSS).^39^ An overview of the statistical decomposition of the virtual library, which contained 199 498 unique quinone-based molecules, is shown in Table 1. The structures of the complete family of 170 CQSs, including their 2D structure drawings and SMILES representations, as well as the total number of *R*-group functionalized derivatives per CQS, are given in ESI Table S1.†

After generating the virtual library, an automated search for finding the commercially available (CA) molecules was performed using an in-house developed tool on the ZINC database, which contains vendor information data on 750 million purchasable compounds.^35^ This tool uses SMILES strings of molecules as inputs and collects the available vendor information from the ZINC database. The search resulted in matching records for 245 organic compounds, which represents a tiny fraction (0.13%) of the grand virtual library that has been built in the current work. This indicates a vast chemical space of molecules that can possibly be synthesized and explored for application as quinone-based cathode materials in LIBs.

### 2.2. Synthetic complexity prediction

While HTCS studies report numerous promising new compounds, their synthesizability is a crucial aspect that is often overlooked during screening. This is partly due to the lack of reliable and rigorous descriptors of synthesizability of organic compounds. In an attempt to address this crucial aspect, we calculated the SCScore, a recently proposed metric by Coley *et al.*,^40^ which provides an assessment of not directly the synthesizability but the synthetic complexity of organic molecules. The model to assign the SCScore was developed by training an artificial neural network on nearly 12 million known chemical reactions. It accepts the SMILES string of a molecule as the input and returns its predicted SCScore on a continuous scale from 1 to 5, where a high SCScore indicates high complexity (and therefore, by proxy, low synthesizability) of precedent synthetic routes for a compound, and *vice versa*.

### 2.3. Redox potential prediction

The redox potentials of quinone-based molecules were predicted at the SEQM-level of theory for the entire virtual library and at the DFT-level for a select group of 641 molecules. At first, the lowest-energy conformers of all the molecules in the gas phase were pre-optimized with the OPLS3e^41^ force field using the MacroModel^42^ module in SMSS. Next, geometry optimization (OPT) and single-point-energy (SPE) calculations were performed for the whole library at the SEQM-level with the AM1 method default parameters.^43^ For these SEQM calculations, the MOPAC2016 (ref. 44) software package as implemented in SMSS was employed. Based on the earlier recommendations for the choice of methods,^45^ we used the lowest unoccupied molecular orbital (LUMO) energies of the quinone reactant molecules that have been optimized at the AM1-level as a descriptor for predicting the redox potentials of all the molecules in the virtual library. Accordingly, the applied linear regression equation is given as:1
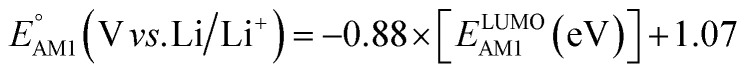
where 
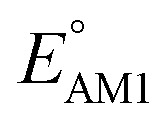
 and 
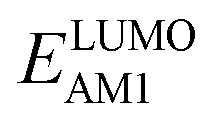
 denote the predicted redox potential and LUMO energy of quinone-based molecules computed at the SEQM-level, respectively. For a group of 641 down-selected candidates, further OPT and SPE calculations were performed at a higher DFT-level of accuracy using the B3LYP functional^46,47^ with the LACVP^++^**^48^ basis set with diffuse and polarization functions. The DFT calculations were performed using the Jaguar program^49^ as implemented in SMSS. The change in energy and RMS density matrix were separately converged to within 5.0 × 10^−5^ and 5.0 × 10^−6^ Hartree, respectively. A ‘medium’ grid density was chosen for performing OPT, whereas a ‘fine’ grid density was used for SPE calculations. Similar to the above, the DFT-calculated LUMO energy of the optimized quinone reactant molecule was employed to predict redox potential. Meanwhile, it has been reported that the electronic structure, particularly the average electric potential of carbon atoms in carbonyl-equipped rings, demonstrates a linear correlation with the redox potential of quinone molecules.^17^ Here, we compared the prediction performance of the two chemical descriptors, and the fitting results are shown in Fig. S1.† It can be observed that the LUMO energy of the quinone molecule (*R*^2^ = 0.97) shows better prediction performance than the average electric potential of carbon atoms in carbonyl-equipped rings (*R*^2^ = 0.81). Similarly, the linear regression equation is given as:2
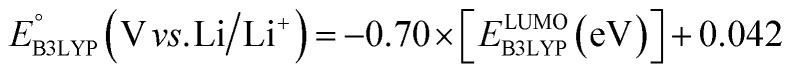
where 
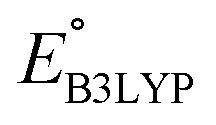
 and 
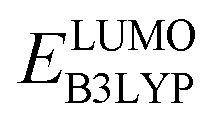
 denote the predicted redox potential and LUMO energy of quinone-based molecules computed at the DFT-level, respectively. It should be noted that both eqn (1) and (2) are valid for an electrolyte formulation of 1 M LiPF_6_ with a (w/w = 1 : 1) solution mixture of ethylene carbonate (EC) and dimethyl carbonate (DMC).^50^

### 2.4. Gravimetric charge capacity and energy density calculation

Another primary metric of electrochemical performance of cathodes is their *Q*, which represents the number of charge carriers per unit mass of active cathode material. While the real value of *Q* for any cathode material will depend on its actual structural composition, molecular packing, and thus determined density, a theoretical upper limit of *Q* can be defined as:^51^3
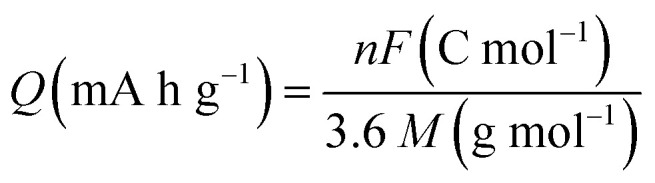
where *M* is the molecular weight of the quinone molecules in g mol^−1^, *n* is the number of transferred e^−^, and *F* is the Faraday constant. For quinone-based molecules in the current work, we assumed that *n* is equal to the number of carbonyl groups in a CQS. The molecular weights were calculated using the SMILES as inputs in SMSS. Similarly, the *W* of organic cathode materials is defined as:^51^4
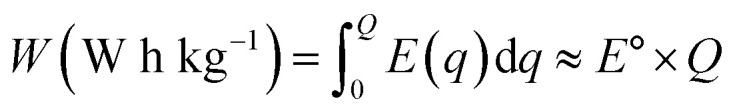
where *E*° represents the average redox potential of the cathode material with respect to Li/Li^+^. The molecular dataset containing the calculated *Q* and *W* values has been provided in the ESI† in CSV format.

### 2.5. Molecular stability prediction

The chemical stability of quinones is a widely debated topic, not only because of their use as electrode materials^27,30^ but also as redox active materials in flow batteries.^24,26,52^ The mechanisms that cause chemical instability in quinones differ not only depending on their molecular structure but also on the electrochemical environment of the surrounding electrolyte, the charge–discharge potentials and rates, and impurities. For use as electrode materials, the change in internal bond lengths upon (de)lithiation is one of the important descriptors of chemical stability because significant bond changes in redox-active molecules are likely to result in instability due to geometrical and mechanical deformations of the electrode.^24,53–57^ Apart from these, structural deformation upon electron transfer is strongly correlated to the molecule's reorganization energy,^26^ which affects the (de)lithiation kinetics.

In the current study, to scrutinize the structural changes upon lithiation, we calculated the root mean square deviation (RMSD) between the reactant and product molecules for all the down-selected 641 candidates. For this task, we employed the LS-align algorithm,^58^ which is an iterative and heuristic tool for atom-level structural comparisons of the molecules. We used the DFT-level optimized reactant and product molecular geometries for the comparisons. To ensure consistency over the explored space of candidate molecules, the calculation of RMSD was based only on the carbon backbones of the pre- and post-lithiated CQSs.^65^ The backbone carbon atoms were selected not only because they represent proportionally the largest atomic fraction count in quinone backbones but also because they undergo significant aromatization-driven changes due to lithiation. It must be noted that the inclusion of the contribution of non-core atoms to RMSD is anyway likely to be erroneous in the gas phase approximation that has been adopted here. This is because the reorganization of these peripheral atoms are highly likely to be affected by the inter-molecular interactions in the solid phase.

### 2.6. Chemical space visualization

We used ChemPlot^59^ to effectively visualize the final screened set of 349 molecules for further analysis and selection of the best candidates for experimental validation. ChemPlot includes three types of dimensionality reduction methods to convert high-dimensional data into visually interpretable two-dimensional plots. A motivation here for applying the dimensionality reduction methods was to find molecules for experimental validation that are structurally ‘different’ from molecules that have previously been investigated in the literature. To start with, we classified the molecules in the set of 349 as either already-investigated, CA or low-SCScore. We used the UMAP^60^ and tailored-similarity methods, both as implemented in ChemPlot, for visual clustering of the 349 molecules.

It is important to note that some CQSs, such as BQ,^26,27^ NQ,^29^ AQ,^29–34^ PQ,^17,29,34^ and several others^18,61,62^ found in our screening library, have been investigated in recent literature as cathode materials for LIBs. Thus, these molecules were classified as already-investigated. After excluding the already-investigated molecules from the screened dataset of 349 molecules, the rest of the molecules that were commercially-available were classified as CA otherwise as low-SCScore. To utilize ChemPlot, we fed it a two-column dataset comprising the SMILES representation of molecules in the first column and their corresponding three categorical target values (low-SCScore, CA, and already-investigated) in the second column. ChemPlot's tailored similarity approach works by initially computing all available descriptors for each molecule, which are then collated into a matrix format, with the rows representing the individual molecules and the columns representing their respective descriptors. The software then identifies which calculated descriptors are correlated with the categorical target values. Finally, the resulting matrix of selected descriptors is utilized as an input for the dimensionality reduction phase.

### 2.7. Experimental validation

The electrodes for the electrochemical measurements were fabricated by mixing the active material 1,4,9,10-anthracenetetraone (AT) with super P and polyvinylidene difluoride (PVDF, average M 275 000 g mol^−1^, Sigma-Aldrich) with a mass ratio of 8 : 1 : 1. In particular, the organic active material was ground with super P in an agate mortar for 15 minutes to obtain the black mixed powder, which was subsequently dispersed in *N*-methylpyrrolidone (NMP, ≤99%, Sigma-Aldrich) solution with the dissolved PVDF (0.05 g ml^−1^). The mixed slurry was treated by ultrasonication for 30 minutes and cast on Al foil. The half-cell consisted of a working electrode, lithium metal (99.9%, Sigma-Aldrich), and a glass fiber separator (ECC1-01-0012-B/L, EL-CELL), while the cell assembly was performed in an argon-filled glove box. The electrolyte is composed of a solution mixture of EC and DMC (v/v = 1 : 1) with 1.0 M LiPF_6_ (Sigma-Aldrich, battery grade). The cyclic voltammetry (CV) measurements were conducted using a galvanostat/potentiostat (VMP-300, BioLogic) with a scan rate of 0.2 mV s^−1^.

## 3. Results and discussion

### 3.1. Metric distribution and HTCS of quinones

**Fig. 2:**
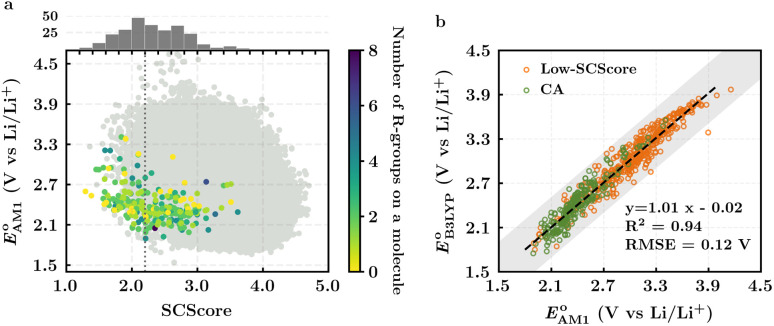
(a) 2D scatter plot showing the distribution of molecules according to their AM1-predicted redox potential 
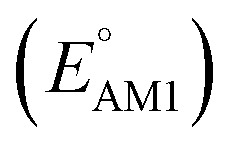
*versus* synthetic complexity score (SCScore) values. The molecules of the virtual library are represented by grey dots, whereas the 245 CA molecules are highlighted with colored dots. The color bar on the right indicates the use of different colors in relation to the number of functional groups that are present on the CA molecules. The histogram at the top depicts the distribution of SCScore values for the CA molecules. The dotted vertical line indicates the med(SCScores) of the CA molecules. (b) Linear correlation of redox potential values for 641 molecules, which have been predicted by using AM1 
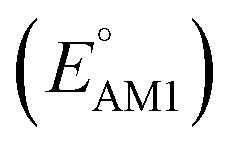
 and DFT-B3LYP 
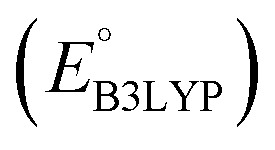
 methods. The green and orange circles denote the CA and low-SCScore molecules, respectively.

The scatter plot of the predicted SCScores and 
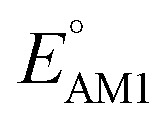
 values of molecules in the virtual library is shown in Fig. 2a. Each grey dot on the map represents a unique molecule, while the entire group of 245 CA molecules is shown using a spectrum of colors, where the colors for molecules were assigned according to the total number of *R*-groups accommodated on them. As can be observed, there is a wide-ranging distribution of molecules across the two metrics with no apparent correlation. The CA molecules are found to accumulate on the bottom-left of the distribution map. Remarkably, with med(SCScores of CA molecules) = 2.2, 95% of the CA molecules had an SCScore < μ(SCScores of all molecules). In addition, the SCScore distribution of molecules is observed to be positively biased towards the molecules that accommodate a small number of *R*-groups. This is expected since the synthesis of molecules that contain a large number of functional groups will be more complex. All these observations support both the aptness of the SCScore as a first-order metric for describing the synthesizability of the virtual molecules and the screening of these molecules on the basis of their SCScores.

The predicted redox potentials of the CA molecules were found to be in the lower range, as shown in Fig. 2a. 180 out of 245 CA molecules had 
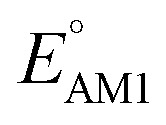
 values of 2.5 ± 0.2 V *vs.* Li/Li^+^. On a more detailed inspection, it is found that a majority (∼81%) of the CA molecules were the functionalized derivatives of only four different quinones, namely, BQ (24 molecules; ∼13%), NQ (43 molecules; ∼24%), AQ (63 molecules; ∼35%), and PQ (16 molecules; ∼9%). In previous experiments, the 
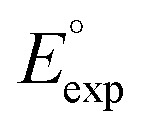
 values of NQ and AQ were between 2.3 and 2.5 V *vs.* Li/Li^+^, whereas PQ had 
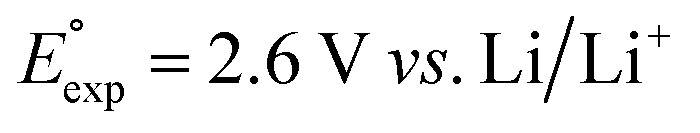
.^29,50,61^ Next, molecules that have both a low-SCScore (≤2.2) and small number of functional groups (≤4) were down-selected for further screening. This resulted in 396 low-SCScore molecules from the library that were not CA. These molecules had 1.90 < 
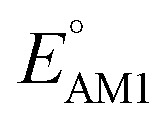
 < 4.25 (±0.03) *vs.* Li/Li^+^, which is a wider potential window than has previously been exercised with quinone-based materials at LIB cathodes.^45^ Given the large variety of functional groups available in the chemical literature, we surmise that the synthesis of quinones with targeted redox potentials is unlikely to be a bottleneck.

Next, we grouped together 245 CA and 396 low-SCScore molecules, thus reaching a total of 641 molecules, for further screening. It has previously been shown that DFT is more accurate than SEQM when predicting the redox potentials of quinones, with the normalized root-mean-square errors (NRMSE) being 12.65% and 16.10%, respectively.^45^ In order to proceed further with higher accuracy, DFT calculations have been performed to recalculate the redox potentials of the 641 molecules. As a side note, the parity plot comparing SEQM (AM1) data against DFT (B3LYP) data is shown in Fig. 2b. The observed RMSE and coefficient of determination (*R*^2^) of 0.12 V *vs.* Li/Li^+^ and 0.94, respectively, imply that the AM1 method is sufficiently accurate when used for the purpose of the initial ranking of molecules in a large virtual library.

**Fig. 3:**
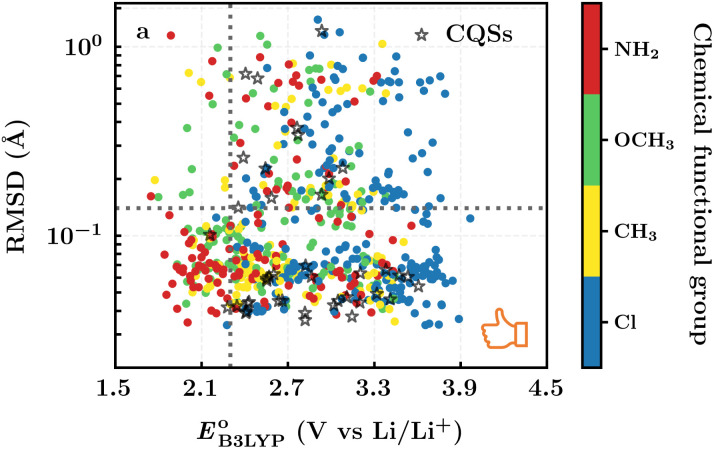
The distribution of calculated RMSD and 
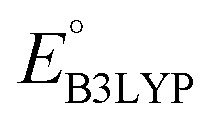
 values for the screened group of 641 molecules. The colors denote the type of *R*-groups, as indicated with the bar on the right, that have been used for the functionalization of the molecules. The black stars denote the core quinone structures (CQSs). The horizontal dotted line marks the upper limit of RMSD values; whereas the vertical dotted line marks the lower limit of 
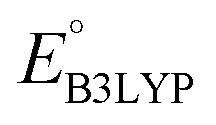
 values that were applied over the screening process. Accordingly, the molecules that satisfied both criteria are located at the bottom-right of the plot.

To evaluate the 641 molecules based on their structural stability and reaction kinetics, we calculated the RMSD of backbone carbon positions between the DFT-optimized structures of pre- and post-lithiated molecules. Fig. 3 shows the distribution of calculated RMSD values of molecules against 
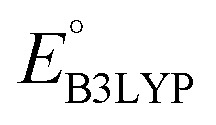
. We found no clear correlations between the calculated RMSD values and either of 
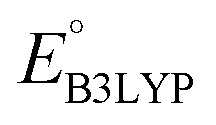
 or chemical functional group type. Nevertheless, it is observed that the molecules functionalized with –Cl groups tend to have higher RMSD (above the horizontal dotted line: ∼11% of 641 molecules for Cl, ∼6% for CH_3_, ∼7% for OCH_3_, and ∼5% for NH_2_), thus making them more likely to undergo degradation. Drawing any inferences on the stability of candidate molecules based on RMSD analysis needs validation against experimentally backed metrics. However, such analysis is currently not possible due to the lack of such corroborating experimental data in the literature. Still, we recommend candidates with low RMSD values for experimental validation. Theoretically, the lengths of single, aromatic, and double bonds between the two neighboring carbon atoms in a molecule are 1.54, 1.40, and 1.33 Å, respectively.^63^ Thus, as a crude approximation, we posit a maximum tolerable value of change in the bond length for an aromatic bond as 0.14 Å (*i.e.* single-aromatic) because bond length changes larger than 0.14 Å will likely be more prone to structural deformation and degradation. Subsequently, applying this criterion to the group of 641 molecules, which is demarcated by the horizontal line in Fig. 3, 430 promising molecules, which included 216 CA molecules, were down-selected.

Continuing the down-selection process, we considered the predicted redox potential values as a metric. The most extensively investigated quinone, both in experiments and computations, is AQ.^29–34^ The calculated 
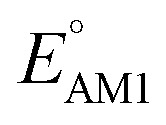
 and 
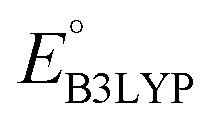
 values for AQ were 2.30 and 2.28 V *vs.* Li/Li^+^, respectively, which are both consistent with the experimentally reported value of 2.27 V.^50,61^ Targeting molecules with a redox potential higher than AQ, 349 of 430 molecules were found to have 
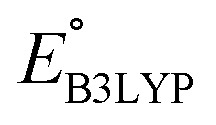
 ≥ 2.28 V *vs.* Li/Li^+^, as demarcated by the vertical line in Fig. 3. This filtered set was found to be composed of 55 distinct CQSs, and it included 140 CA molecules. These encouraging findings indicate a wealth of diverse, purchasable and potentially high-performing molecules which are worthy of in-depth analysis aimed at digging out good cathode materials for LIBs. A CSV file containing comprehensive information on each of the 349 molecules can be found in the Data availability section.

**Fig. 4:**
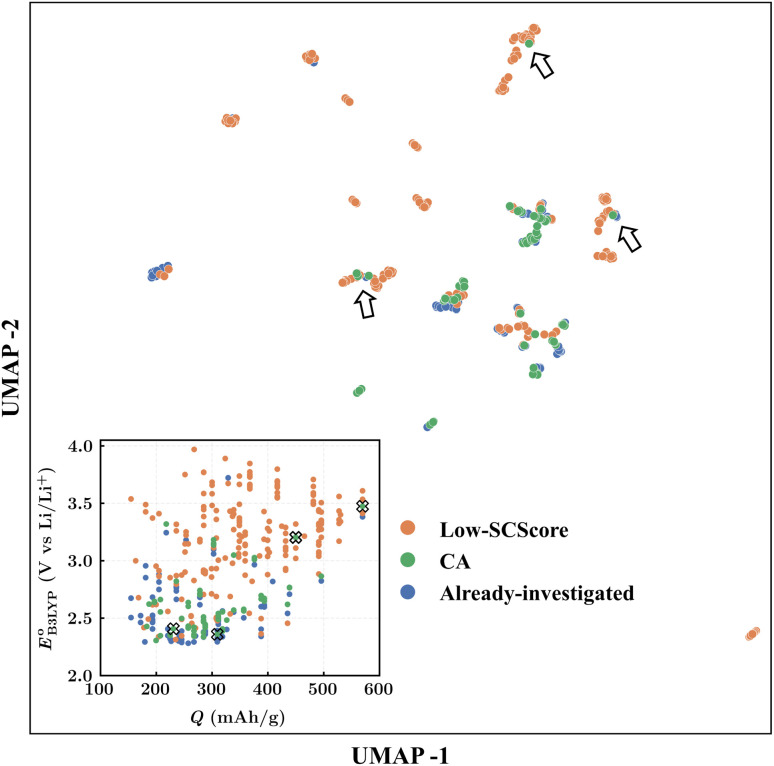
The ChemPlot-visualized dimensionally reduced chemical space of the 349 candidate molecules. The inset shows the distribution of molecules with respect to the predicted values of redox potential 
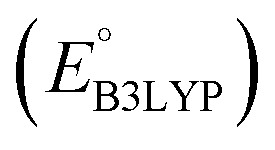
 and gravimetric charge capacity (*Q*). Black arrows and black crosses point to the same molecules that are shown by the two different visualization methods, respectively in the main and inset plots.

Finally, to determine the molecules for experimentation in the current study, we visualized the clustering of the 349 quinones by using ChemPlot (Fig. 4). The principle that guides the use of ChemPlot is to identify molecules that are both intriguing for further validation and characterization, while also being structurally distinct from the molecules that have already been studied in the literature, considering that there are numerous molecules available to choose from.

The Fig. 4 inset shows the distribution of 
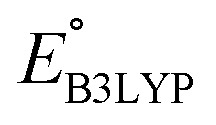
 and *Q* of the 349 molecules. As can be observed in Fig. 3 and 4, there are ample possibilities in terms of a multitude of properties, including redox potential, stability, charge capacity and chemical variety in functional groups. Quite remarkably, it is apparent from the Fig. 4 inset that the CA (green dots) and already-investigated (blue dots) molecules tend to show only modest energy-related properties in comparison to the virtual library molecules with low SCScores (orange dots). The CA and already-investigated molecules were distributed in the low 
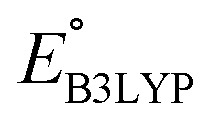
 and *Q* regions. Interestingly, many of the already-investigated molecules were located in proximity to the CA molecules. Consequently, these molecules were not prioritized for experimental validation in the current study, as our main interest is in molecules that have not been studied for LIB cathodes.

As the prime candidates for our experiments, we focused on the CA molecules that were located in the same clusters as a large number of low-SCScore molecules. Accordingly, five CA quinones were identified in three different clusters of low SCScore virtual molecules that are pointed out using the black arrows in Fig. 4. The same five CA molecules are also highlighted using the black crosses in the inset figure of Fig. 4. The calculated cathode-related properties of these five CA molecules are summarized in Table 2. The molecules #1 and #5 are predicted to exhibit lower cathode-related performance than the rest of the three molecules based on the calculated properties of 
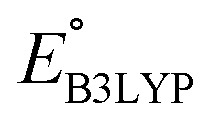
, *Q*, and *W*. Thus, we prioritized molecules #2, #3 and #4 for experimentation and among them we selected molecule #4 (AT) for electrochemical characterization based on its in-stock commercial availability.

**Table 2:** 2D structural representations, predicted redox potential 
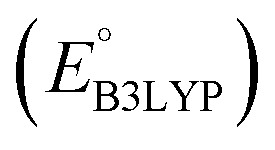
, gravimetric charge capacity (*Q*), and gravimetric energy density (*W*) of the top five CA quinones for LIB cathodes

#	2D structures	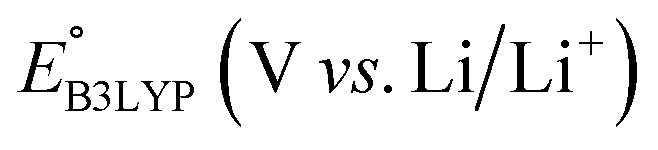	*Q* (mA h g^−1^)	*W* (W h kg^−1^)	Available in-stock
1	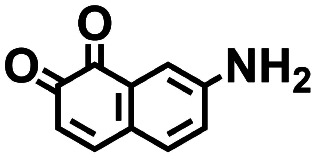	2.36	309	729	No
2	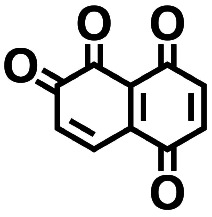	3.47	570	1978	No
3	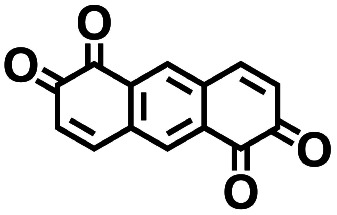	3.20	450	1440	No
4	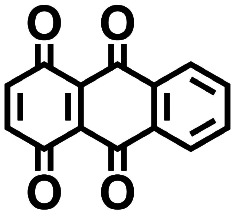	3.20	450	1440	Yes
5	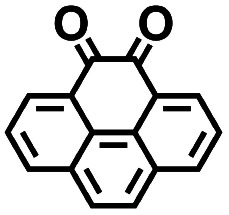	2.41	231	557	Yes

### 3.2. Experimental validation

**Fig. 5:**
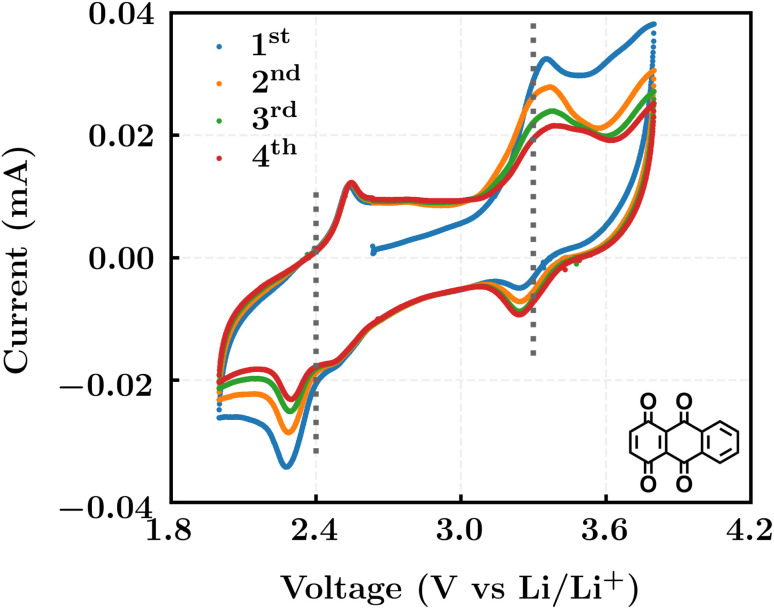
Cyclic voltammograms in the first four cycles of 1,4,9,10-anthracenetetraone (AT) in 1 mol L^−1^ LiPF_6_ and a v/v = 1 : 1 solution mixture of EC and DMC.

We investigated the electrochemical lithiation behavior of AT, which is a readily purchasable top candidate molecule that has been identified *via* HTCS as explained above. We performed CV analysis of the candidate compound in a half cell against lithium metal. The open circuit voltage of the half-cell containing the initial electrode with a pristine active organic material was measured to be approximately 2.6 V, which indicates its promising nature as an electrode for LIBs. As shown in Fig. 5, subsequent CV cycling in the range between 2.0 and 3.8 V clearly demonstrated the presence of redox peaks indicating the (de)lithiation reactions within the half cell. Repetitive cycling within this voltage range showed a decrease/increase in the peak currents within the first four cycles. However, very minimal changes in the peak voltage positions were observed from the first cycle to the fourth cycle. The experimental data exhibited clearly visible reduction peaks at 2.28 V and 3.25 V and corresponding oxidation peaks at respectively 2.55 V and 3.35 V. Thus, the averages of redox potentials for this compound were 2.4 and 3.3 *vs.* Li/Li^+^, as shown with vertical dotted lines in Fig. 5. The high redox potential value, which implies the reaction of a pristine compound with two Li atoms, was in good agreement with our predicted values that have been obtained by using both the AM1 
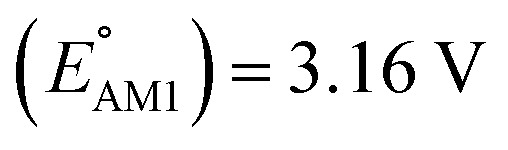
 and the B3LYP 
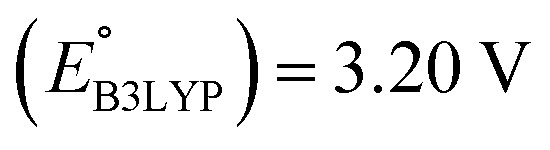
 methods (Table 2). For the low redox potential, we used the computed LUMO+1 energy of AT 
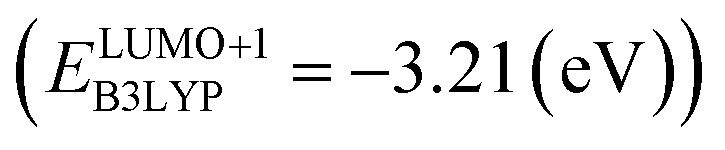
 and eqn (2) to obtain the predicted 
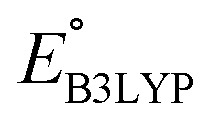
 between AT-Li_2_ and AT-Li_4_. The corresponding calculated value of 2.29 V was close to the experimental value of 2.4 V.

### 3.3. Structure–property relationships

To facilitate the rational design of high-performance quinone-based cathode materials beyond the current work, we investigated the effects of structural features of molecules, including the type of functional groups, number of aromatic rings in core structures, and type of carbonyl groups, chiefly on the two key predicted properties of *E*° and SCScore.

**Fig. 6:**
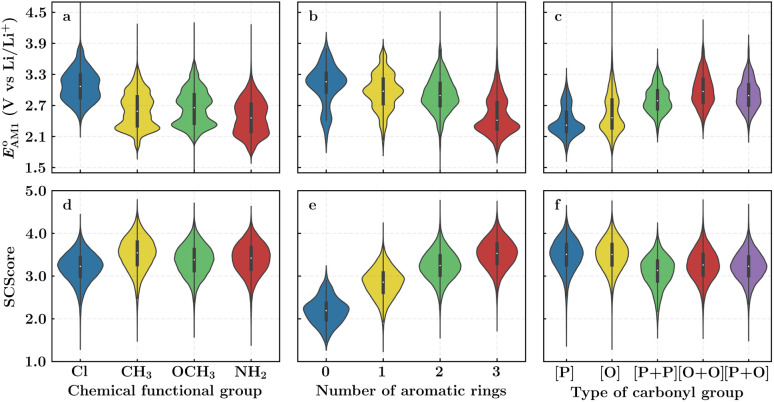
Violin plots showing distributions of 
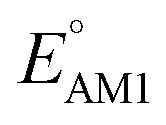
 (a–c) and SCScore (d–f) data for approximately 200k candidates found in the virtual library. The distributions are shown with respect to: (a and d) the type of functional group, (b and e) the number of aromatic rings, and (c and f) type of carbonyl groups that were present in molecules. The white dots that are in the center of black bars represent the median values.

The effects of functional groups on 
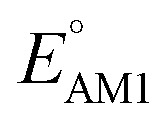
 and SCScore are shown in Fig. 6a and d, respectively, and the more detailed distributions in terms of the different total number of rings (∼size) are shown in Fig. S2.† It is found that the presence of highly electron-withdrawing functional groups (–Cl > –CH_3_ > –OCH_3_ > –NH_2_) on molecules shifts the predicted redox distribution towards higher potentials (Fig. 6a and S2†). On the other hand, as shown in Fig. 6d, the SCScore was the lowest for –Cl functionalized molecules, but it had no clear dependence on the choice of the functional group otherwise. Also observable in Fig. S2† is the fact that the effect of functional groups on 
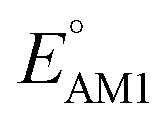
 and SCScore is consistent across the different groups of total rings. The potential window of one-ring quinones is quite narrow, while the redox potentials of other size groups span a much broader range. Surprisingly, there are hardly any differences in the potential range spanned by two-ring, three-ring, and four-ring quinone groups despite having very different number positions available for functionalization. The detailed distributions in Fig. S2† also show that the SCScore increases with an increasing number of total rings.

The number of aromatic rings, which is essentially the number of rings that include no carbonyl groups of a reactant molecule CQS, also had an influence on both properties (Fig. S3†). An increase in the number of aromatic rings in reactant molecules resulted in shifts towards lower redox potentials (Fig. 6b) and higher SCScores (Fig. 6e). This anti-correlation implies that it will be difficult to independently tune the redox potential and the synthesizability of molecules just by varying the number of aromatic rings. It is also observed that the ranges over which both 
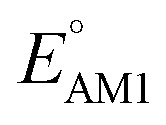
 and SCScore vary also expand with the number of aromatic rings found on molecules. This is not so surprising, as molecules with a large number of rings will have more atomic node positions available for functionalization and they are therefore more likely to show a high degree of structural and electrochemical tunability.

Lastly, we analyzed the effects of the type and number of carbonyl groups on 
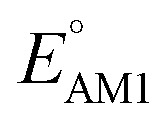
 and SCScore. The distribution of redox potentials, with respect to the type of carbonyl groups, is shown in Fig. 6c, and the detailed information in terms of the total number of rings is shown in Fig. S3.† More detailed 2D histograms of molecules having different carbonyl groups are shown in Fig. S4–S7.† For all the molecules that had the same number of carbonyl groups, the ones that contained 1,2-benzoquinone, [O], spanned a wider range of redox potentials when compared to the ones that contained 1,4-benzoquinone, [P]. Furthermore, it should be noted that [P] and [O] have a quite similar distribution of 
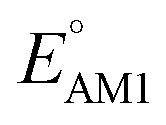
 for one-ring molecules as shown in Fig. S3.† As the total number of rings increases, [O] shows a wider potential window and higher 
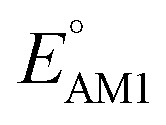
 than [P]. It can also be observed in Fig. S3† that the distributions of 
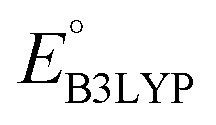
 show little difference for [P + P], [O + O] and [P + O] conformers in two-ring quinones. For the three-ring and four-ring quinones, [O + O] and [P + O] conformers have relatively higher 
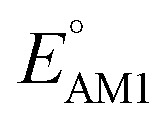
 than [P + P] conformers. Interestingly, the molecules with two pairs of carbonyl groups showed higher redox potentials, which also motivated our choice of the AT compound for the CV experiments as discussed above. The [O + O] group of molecules achieved the highest median redox potential among all the molecules with four carbonyl groups. This observation can be attributed to favorable energetic interactions between a Li atom and two C=O groups at neighboring positions on the same ring, thus resulting in a significantly higher stabilization of the product compound. A similar trend was also pointed out by Poizot *et al.*, in which they observed that *ortho*-quinones can achieve a redox potential about 300 mV higher than *para*-quinones.^64^ The overall influence of carbonyl groups on pushing the redox potential of the molecules to higher values can be summarized as follows: [O + O] ≥ [P + O] > [P + P] > [O] > [P]. As shown in Fig. 6f, the SCScore metric showed a relatively weak correlation with the type of carbonyl group present, although quinones containing two pairs of carbonyl groups had marginally lower SCScores compared to those containing a single pair. Fig. S3† serves as further evidence that an increase in the number of rings present in quinone reactants leads to a higher SCScore.

## 4. Conclusions

In this study, a hierarchical high-throughput computational screening has been designed to identify new cathode candidates from a chemical space of 199 498 quinone-based molecules for LIBs. To rank the molecules based on their synthesizability, the SCScore metric of each compound was evaluated using a ML-based model, where a low SCScore indicated a high possibility of synthesizability. In parallel, a search for vendor information for the virtually designed molecules was performed in the ZINC database, which revealed the commercial availability records for 245 unique molecules. Interestingly, most of the commercially available molecules were found to have low SCScores, thus validating the usefulness of this metric. Subsequently considering molecules that have low SCScores and a small number of functional groups, 396 additional virtual molecules that had no ZINC records were down-selected from the screening library. Thus, a cumulative number of 641 candidate molecules were subjected to DFT calculations for further validation. Next, an RMSD analysis of the DFT-calculated structural distortions, due to lithiation, was applied to evaluate the propensity of these 641 molecules to structural degradation. Consequently, 349 quinone-based molecules were identified that had low RMSD and high 
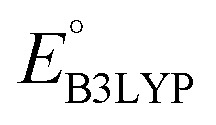
 values. After the use of dimensionality reduction and tailored similarity methods for chemical space exploration, we targeted one readily purchasable molecule, AT, for validation. This compound showed highly promising redox features in CV experiments. The approach presented in the current study resulted in a collection of top-candidates that are not only predicted to-be high-performing battery materials but are also deemed to be synthetically accessible, which together make them attractive for further investigations. Finally, the accompanying calculation dataset of molecules is anticipated to be a useful resource as both a reference and a training data source for the development of new materials and predictive models, respectively.

## Data availability

The computed properties of 641 molecules have been compiled in a data frame which contains information, including SMILES representations, molecular formula, molecular weight, *Q*, 
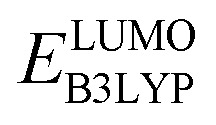
, 
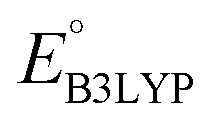
, W, SCScore, and RMSD values, and commercial vendor page retrieved from the ZINC database whenever available. This data frame is available as a self-contained ESI† in CSV format.

## Author contributions

Conceptualization, S. E.; methodology, X. Z., A. K. and S. E.; modeling and validation, X. Z. and A. K.; formal analysis and discussion, X. Z., A. K., R. A. J. J. and S. E.; experiment and validation, J. Z. and M. H.; writing – original draft preparation, X. Z. (computational part) and J. Z. (experimental part); writing – review and editing, X. Z., A. K., M. H., R. A. J. J. and S. E.; supervision, A. K., R. A. J. J. and S. E.; project administration, S. E.; funding acquisition, X. Z. and S. E. All authors have read and agreed to the published version of the manuscript.

## Conflicts of interest

There are no conflicts to declare.

## Supplementary Material

DD-002-D2DD00112H-s001

DD-002-D2DD00112H-s002
