# Supplementary material for: Discovery of lead quinone cathode materials for Li-ion batteries
Source: Digit Discov. 2023 May 30;2(4):1016–25. doi: 10.1039/d2dd00112h (PMC10408572; doi:10.1039/d2dd00112h)
Supplement: DD-002-D2DD00112H-s002 [file DD-002-D2DD00112H-s002.pdf]

Electronic Supplementary Material (ESI) for Digital Discovery.
This journal is © The Royal Society of Chemistry 2023

Supplementary Information

Discovery of lead quinone cathode materials for Li-ion batteries

Xuan Zhou,[a,b], Abhishek Khetan,[a,d], Jie Zheng[e], Mark Huijben,[e], René A.J. Janssen,[a,c], Süleyman Er, [a,*]

[a]*DIFFER – Dutch Institute for Fundamental Energy Research, De Zaale 20, Eindhoven, 5612 AJ, the Netherlands*
[b]*Department of Applied Physics, Eindhoven University of Technology, Eindhoven, 5600 MB, the Netherlands*
[c]*Molecular Materials and Nanosystems, Institute for Complex Molecular System, Eindhoven University of Technology, Eindhoven, 5600 MB, the Netherlands*
[d]*Multiscale Modeling of Heterogeneous Catalysis in Energy Systems, RWTH Aachen University, Aachen, 52062, Germany*
[e]*MESA+ Institute for Nanotechnology, University of Twente, Enschede, 7500 AE, the Netherlands*

*Corresponding author
Email address: s.er@differ.nl (Süleyman Er,)

List of Tables and Figures

Table S1 . . . . S4

Figure S1 . . . . S21

Figure S2 . . . . S22

Figure S3 . . . . S23

Figure S4 . . . . S24

Figure S5 . . . . S25

Figure S6 . . . . S26

Figure S7 . . . . S27

Table S1: 2D molecular drawings, SMILES representations, and the number of functionalized derivatives of the 170 core quinones structures that have been used to build the virtual screening library in the current study.

No.	2D structures	SMILES	Number of functionalized derivatives
1	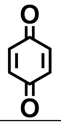	O=C1C=CC(=O)C=C1	6
2	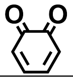	O=C1C(=O)C=CC=C1	9
3	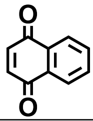	c1cccc(c12)C<br>(=O)C=CC2=O	35
4	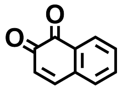	c1cccc(c12)C=C<br>C(=O)C2=O	63
5	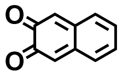	c1cccc(c1=2)=C<br>C(=O)C(=O)C2	35
6	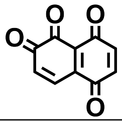	O=c1ccc(=O)c<br>(c12)ccc(=O)c2=O	15
7	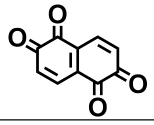	c1cc(=O)c(=O)c<br>(c12)ccc(=O)c2=O	9
8	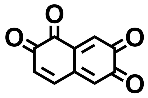	c1c(=O)c(=O)cc<br>(c12)ccc(=O)c2=O	15
9	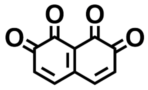	O=c1c(=O)ccc<br>(c12)ccc(=O)c2=O	9
10	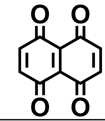	O=c1ccc(=O)c<br>(c12)c(=O)ccc2=O	6
11	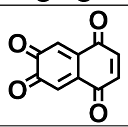	c1c(=O)c(=O)cc<br>(c12)c(=O)ccc2=O	9

Continued on next page

Table S1 – Continued from previous page

No.	2D structures	SMILES	Number of functionalized derivatives
12	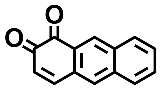	O=C1C(=O)C=Cc<br>(c12)cc3c(c2)cccc3	255
13	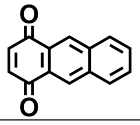	O=C1C=CC(=O)c(c12)<br>cc3c(c2)cccc3	135
14	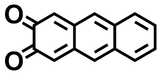	c1cccc(c12)cc=3c(c2)<br>=CC(=O)C(=O)C3	135
15	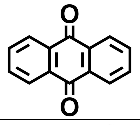	c1cccc(c12)C(=O)c3<br>c(C2=O)cccc3	75
16	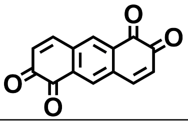	O=c1c(=O)ccc(c12)c<br>c3c(c2)ccc(=O)c3=O	35
17	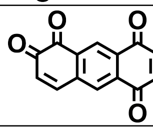	O=c1ccc(=O)c(c12)c<br>c3c(c2)c(=O)c(=O)cc3	63
18	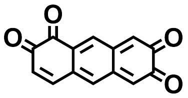	O=c1c(=O)ccc(c12)c<br>c3c(c2)cc(=O)c(=O)c3	63
19	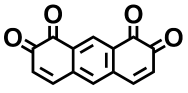	O=c1c(=O)ccc(c12)c<br>c3c(c2)c(=O)c(=O)cc3	39
20	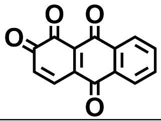	O=c1c(=O)ccc(c12)c<br>(=O)c3c(c2=O)cccc3	63
21	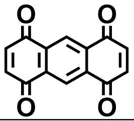	O=c1ccc(=O)c(c12)c<br>c3c(c2)c(=O)ccc3=O	23
22	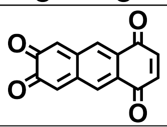	O=c1ccc(=O)c(c12)c<br>c3c(c2)cc(=O)c(=O)c3	35

Continued on next page

Table S1 – Continued from previous page

No.	2D structures	SMILES	Number of functionalized derivatives
23	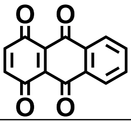	O=c1ccc(=O)c(c12)c(=O)c3c(c2=O)cccc3	35
24	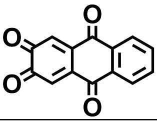	c1c(=O)c(=O)cc(c12)c(=O)c3c(c2=O)cccc3	35
25	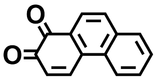	C1=CC(=O)C(=O)c(c1c23)ccc2cccc3	255
26	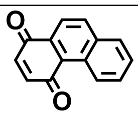	c1cccc(c1c23)ccc2C(=O)C=CC3=O	255
27	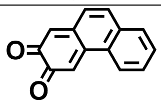	C1C(=O)C(=O)C=c(c=1c23)ccc2cccc3	255
28	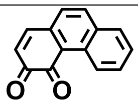	c1cccc(c1c23)ccc3C=CC(=O)C2=O	255
29	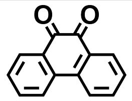	c1cccc(c1c23)C(=O)C(=O)c2cccc3	135
30	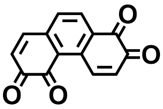	c1cc(=O)c(=O)c(c1c23)ccc3ccc(=O)c2=O	63
31	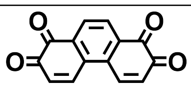	c1cc(=O)c(=O)c(c1c23)ccc2c(=O)c(=O)cc3	35
32	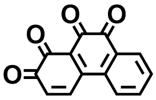	O=c1c(=O)ccc(c12)c3c(c(=O)c2=O)cccc3	63
33	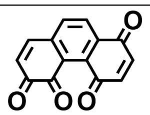	O=c1c(=O)ccc(c1c23)ccc2c(=O)ccc3=O	63
34	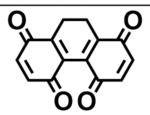	O=c1ccc(=O)c(c1c23)ccc2c(=O)ccc3=O	35

Continued on next page

Table S1 – Continued from previous page

No.	2D structures	SMILES	Number of functionalized derivatives
35	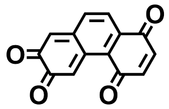	c1c(=O)c(=O)cc(c1c23) ccc2c(=O)ccc3=O	63
36	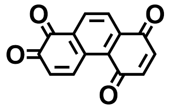	c1cc(=O)c(=O)c(c1c23) ccc2c(=O)ccc3=O	63
37	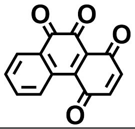	O=c1ccc(=O)c(c12)c (=O)c(=O)c3c2cccc3	63
38	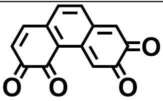	c1c(=O)c(=O)cc(c1c23)c cc3ccc(=O)c2=O	63
39	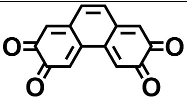	c1c(=O)c(=O)cc(c1c23)c cc2cc(=O)c(=O)c3	35
40	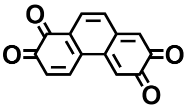	c1cc(=O)c(=O)c(c12)c cc3c2cc(=O)c(=O)c3	63
41	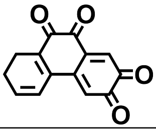	c1cccc(c1c23)c(=O)c (=O)c2cc(=O)c(=O)c3	63
42	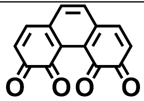	O=c1c(=O)ccc(c1c23)c cc3ccc(=O)c2=O	35
43	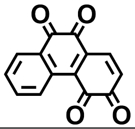	c1cccc(c1c23)c(=O)c (=O)c2ccc(=O)c3=O	63
44	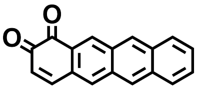	O=C1C(=O)C=Cc(c12)c c3c(c2)cc4c(c3)cccc4	1023
45	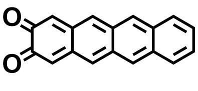	c1cccc(c2)c1cc(c23)c c=4c(c3)=CC(=O)C(=O)C4	527
46	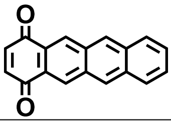	O=C1C=CC(=O)c(c12)c c3c(c2)cc4c(c3)cccc4	527

Continued on next page

Table S1 – Continued from previous page

No.	2D structures	SMILES	Number of functionalized derivatives
47	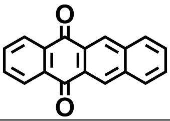	c1cccc(c2)c1cc(c23)C<br>(=O)c4c(C3=O)cccc4	527
48	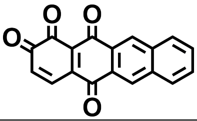	O=c1c(=O)ccc(c12)c(=O)<br>c3c(c2=O)cc4c(c3)cccc4	255
49	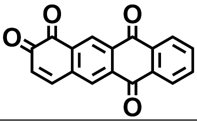	c1cc(=O)c(=O)c(c2)c1cc<br>(c23)c(=O)c4c(c3=O)cccc4	255
50	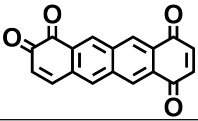	O=c1ccc(=O)c(c12)cc3c<br>(c2)cc4c(c3)ccc(=O)c4=O	255
51	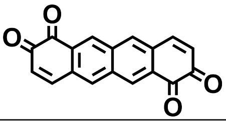	O=c1c(=O)ccc(c12)cc3c<br>(c2)cc4c(c3)c(=O)c(=O)cc4	135
52	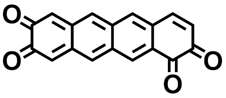	O=c1c(=O)ccc(c12)cc3c<br>(c2)cc4c(c3)cc(=O)c(=O)c4	255
53	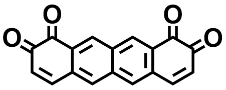	O=c1c(=O)ccc(c12)cc3c<br>(c2)cc4c(c3)ccc(=O)c4=O	135
54	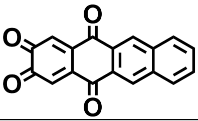	c1cccc(c2)c1cc(c23)c(=O)<br>c4c(c3=O)cc(=O)c(=O)c4	135
55	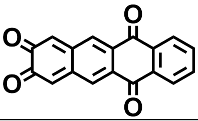	c1cccc(c2=O)c1c(=O)c<br>(c23)cc4c(c3)cc(=O)c(=O)c4	135
56	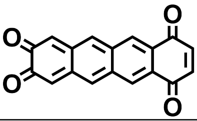	O=c1ccc(=O)c(c12)cc3c<br>(c2)cc4c(c3)cc(=O)c(=O)c4	135
57	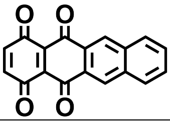	O=c1ccc(=O)c(c12)c(=O)<br>c3c(c2=O)cc4c(c3)cccc4	135

Continued on next page

Table S1 – Continued from previous page

No.	2D structures	SMILES	Number of functionalized derivatives
58	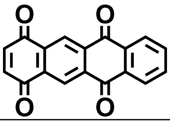	c1cccc(c2=O)c1c(=O)c (c23)cc4c(c3)c(=O)ccc4=O	135
59	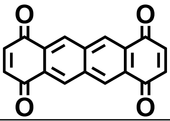	O=c1ccc(=O)c(c12)cc3c (c2)cc4c(c3)c(=O)ccc4=O	75
60	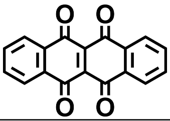	c1cccc(c2=O)c1c(=O)c(c23) c(=O)c4c(c3=O)cccc4	75
61	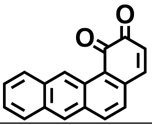	c1cccc(c2)c1cc(c2c34)c cc4C=CC(=O)C3=O	1023
62	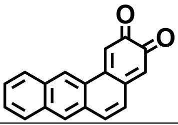	C1C(=O)C(=O)C=c(c=1c23)c cc2cc4c(c3)cccc4	1023
63	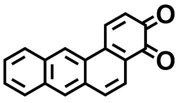	C1=CC(=O)C(=O)c(c1c23)c cc2cc4c(c3)cccc4	1023
64	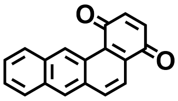	c1cccc(c2)c1cc(c2c34) ccc3C(=O)C=CC4=O	1023
65	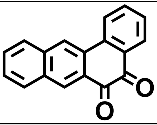	c1cccc(c1c23)C(=O)C(=O) c2cc4c(c3)cccc4	1023
66	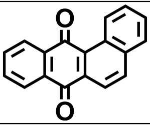	c1cccc(c1c23)ccc2C(=O) c4c(C3=O)cccc4	1023
67	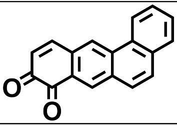	c1cccc(c1c23)ccc2cc4 c(c3)C=CC(=O)C4=O	1023
68	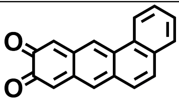	c1cccc(c1c23)ccc2cc=4 c(c3)=CC(=O)C(=O)C4	1023

Continued on next page

Table S1 – Continued from previous page

No.	2D structures	SMILES	Number of functionalized derivatives
69	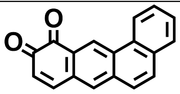	c1cccc(c1c23)ccc2cc4 c(c3)C(=O)C(=O)C=C4	1023
70	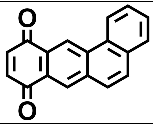	c1cccc(c1c23)ccc2cc4 c(c3)C(=O)C=CC4=O	1023
71	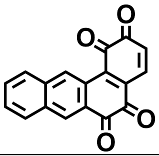	c1cccc(c2)c1cc(c2c34) c(=O)c(=O)c3ccc(=O)c4=O	255
72	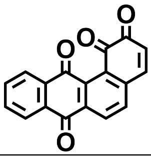	O=c1c(=O)ccc(c1c23)ccc2 c(=O)c4c(c3=O)cccc4	255
73	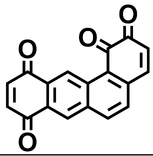	O=c1ccc(=O)c(c2)c1cc (c2c34)ccc4ccc(=O)c3=O	255
74	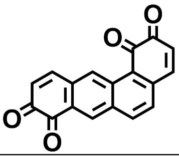	O=c1c(=O)ccc(c2)c1cc (c2c34)ccc4ccc(=O)c3=O	255
75	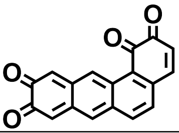	c1c(=O)c(=O)cc(c2)c1cc (c2c34)ccc4ccc(=O)c3=O	255
76	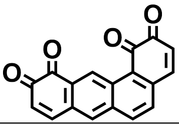	c1cc(=O)c(=O)c(c2)c1cc (c2c34)ccc4ccc(=O)c3=O	255
77	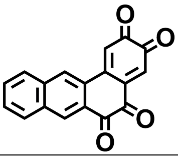	c1c(=O)c(=O)cc(c1c23)c(=O) c(=O)c2cc4c(c3)cccc4	255

Continued on next page

Table S1 – Continued from previous page

No.	2D structures	SMILES	Number of functionalized derivatives
78	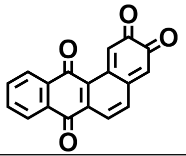	c1cccc(c2=O)c1c(=O)c(c23<br>)ccc4c3cc(=O)c(=O)c4	255
79	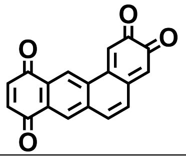	c1c(=O)c(=O)cc(c1c23)<br>ccc2cc4c(c3)c(=O)ccc4=O	255
80	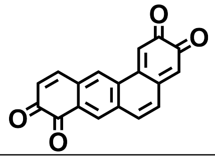	c1c(=O)c(=O)cc(c1c23)<br>ccc2cc4c(c3)ccc(=O)c4=O	255
81	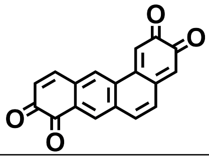	c1c(=O)c(=O)cc(c1c23)<br>ccc2cc4c(c3)cc(=O)c(=O)c4	255
82	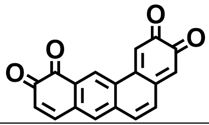	c1c(=O)c(=O)cc(c1c23)<br>ccc2cc4c(c3)c(=O)c(=O)cc4	255
83	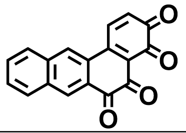	O=c1c(=O)ccc(c12)c3c<br>(c(=O)c2=O)cc4c(c3)cccc4	255
84	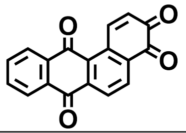	c1cc(=O)c(=O)c(c1c23)<br>ccc2c(=O)c4c(c3=O)cccc4	255
85	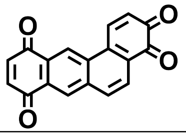	c1cc(=O)c(=O)c(c12)<br>ccc3c2cc4c(c3)c(=O)ccc4=O	255
86	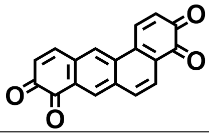	c1cc(=O)c(=O)c(c12)<br>ccc3c2cc4c(c3)c(=O)c(=O)cc4	255

Continued on next page

Table S1 – Continued from previous page

No.	2D structures	SMILES	Number of functionalized derivatives
87	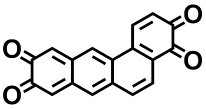	c1cc(=O)c(=O)c2ccc (c3c12)cc4c(c3)cc(=O)c(=O)c4	255
88	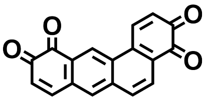	c1cc(=O)c(=O)c(c12)cc c3c2cc4c(c3)ccc(=O)c4=O	255
89	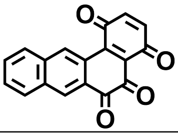	O=c1ccc(=O)c(c12)c(=O) c(=O)c3c2cc4c(c3)cccc4	255
90	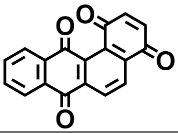	O=c1ccc(=O)c(c1c23) ccc2c(=O)c4c(c3=O)cccc4	255
91	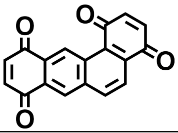	O=c1ccc(=O)c(c2)c1cc (c2c34)ccc3c(=O)ccc4=O	255
92	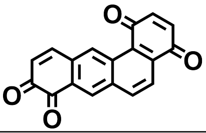	O=c1ccc(=O)c2ccc(c3c12) cc4c(c3)ccc(=O)c4=O	255
93	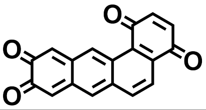	c1c(=O)c(=O)cc(c2)c1c c(c2c34)ccc3c(=O)ccc4=O	255
94	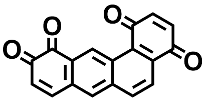	c1cc(=O)c(=O)c(c2)c1c c(c2c34)ccc3c(=O)ccc4=O	255
95	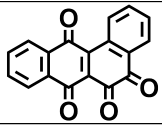	c1cccc(c2=O)c1c(=O)c (c23)c(=O)c(=O)c4c3cccc4	255
96	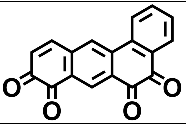	c1cccc(c1c23)c(=O)c(=O) c2cc4c(c3)ccc(=O)c4=O	255
97	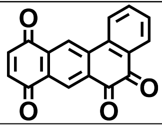	c1cccc(c1c23)c(=O)c(=O) c2cc4c(c3)c(=O)ccc4=O	255

Continued on next page

Table S1 – Continued from previous page

No.	2D structures	SMILES	Number of functionalized derivatives
98	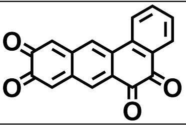	c1cccc(c1c23)c(=O)c(=O)<br>c2cc4c(c3)cc(=O)c(=O)c4	255
99	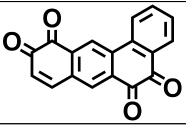	c1cccc(c1c23)c(=O)c(=O)<br>c2cc4c(c3)c(=O)c(=O)cc4	255
100	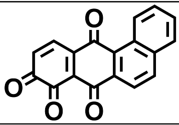	O=c1c(=O)ccc(c2=O)c1c(=O)<br>c(c23)ccc4c3cccc4	255
101	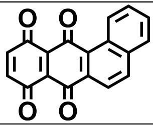	O=c1ccc(=O)c(c12)c(=O)<br>c3c(c2=O)c4c(cc3)cccc4	255
102	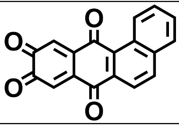	c1c(=O)c(=O)cc(c2=O)<br>c1c(=O)c(c23)ccc4c3cccc4	255
103	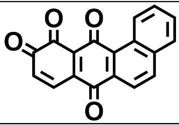	c1cc(=O)c(=O)c(c2=O)<br>c1c(=O)c(c23)ccc4c3cccc4	255
104	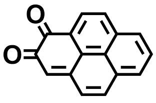	c12c3c4ccc1c(=O)c<br>(=O)cc2ccc3ccc4	255
105	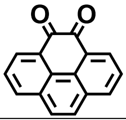	c12c3c4c(=O)c(=O)<br>c1cccc2ccc3ccc4	135
106	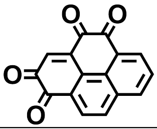	c12c3c4c(=O)c(=O)c1c<br>c(=O)c(=O)c2ccc3ccc4	63
107	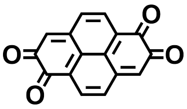	c12c3c4c(=O)c(=O)cc3<br>ccc1c(=O)c(=O)cc2cc4	35
108	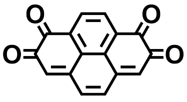	c12c3c4c(=O)c(=O)cc3<br>ccc2cc(=O)c(=O)c1cc4	35

Continued on next page

Table S1 – Continued from previous page

No.	2D structures	SMILES	Number of functionalized derivatives
109	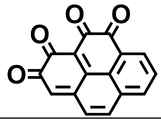	c12c3c(=O)c(=O)c4c1c<br>(ccc4)ccc2cc(=O)c3=O	63
110	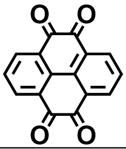	c12c3c4c(=O)c(=O)c1c<br>ccc2c(=O)c(=O)c3ccc4	23
111	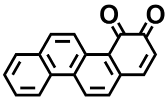	c1cccc(cc2)c1c(c2c34)<br>ccc4C=CC(=O)C3=O	1023
112	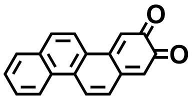	C1C(=O)C(=O)C=c(cc2)<br>c=1c(c23)ccc4c3cccc4	1023
113	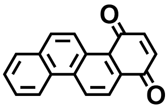	c1cccc(cc2)c1c(c2c34)<br>ccc3C(=O)C=CC4=O	1023
114	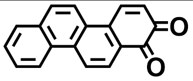	C1=CC(=O)C(=O)c(cc2)<br>c1c(c23)ccc4c3cccc4	1023
115	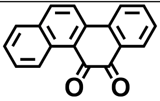	c1cccc(C(=O)C2=O)c1c<br>(c23)ccc4c3cccc4	1023
116	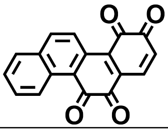	c1cccc(cc2)c1c(c2c34)<br>c(=O)c(=O)c3ccc(=O)c4=O	255
117	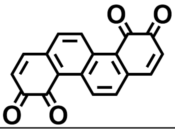	O=c1c(=O)ccc(cc2)c1<br>c(c2c34)ccc4ccc(=O)c3=O	135
118	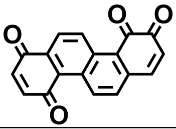	O=c1c(=O)ccc(cc2)c1<br>c(c2c34)ccc3c(=O)ccc4=O	255
119	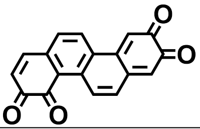	c1c(=O)c(=O)cc(cc2)c1<br>c(c2c34)ccc4ccc(=O)c3=O	255

Continued on next page

Table S1 – Continued from previous page

No.	2D structures	SMILES	Number of functionalized derivatives
120	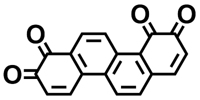	c1cc(=O)c(=O)c(cc2)c1c(c2c34)ccc4ccc(=O)c3=O	255
121	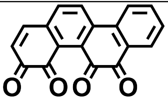	O=c1c(=O)ccc(c1c23)ccc2c4c(c(=O)c3=O)cccc4	255
122	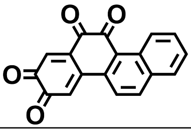	c1c(=O)c(=O)cc(c(=O)c2=O)c1c(c23)ccc4c3cccc4	255
123	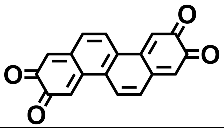	c1c(=O)c(=O)cc(cc2)c1c(c23)ccc4c3cc(=O)c(=O)c4	135
124	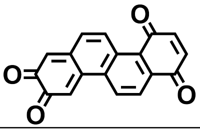	c1c(=O)c(=O)cc(cc2)c1c(c2c34)ccc3c(=O)ccc4=O	255
125	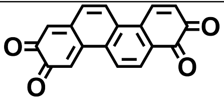	c1cc(=O)c(=O)c(c1c23)ccc2c4c(cc3)cc(=O)c(=O)c4	255
126	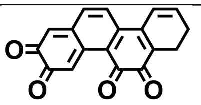	c1cccc(c(=O)c2=O)c1c(c23)ccc4c3cc(=O)c(=O)c4	255
127	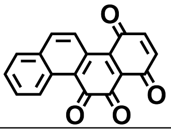	O=c1ccc(=O)c(c12)c(=O)c(=O)c3c2ccc4c3cccc4	255
128	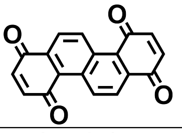	O=c1ccc(=O)c(cc2)c1c(c2c34)ccc3c(=O)ccc4=O	135
129	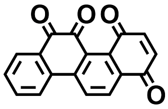	O=c1ccc(=O)c(c1c23)ccc2c4c(c(=O)c3=O)cccc4	255
130	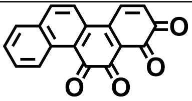	c1cc(=O)c(=O)c(c(=O)c2=O)c1c(c23)ccc4c3cccc4	255

Continued on next page

Table S1 – Continued from previous page

No.	2D structures	SMILES	Number of functionalized derivatives
131	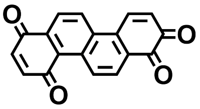	c1cc(=O)c(=O)c(cc2)c1c (c2c34)ccc3c(=O)ccc4=O	255
132	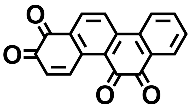	c1cccc(c(=O)c2=O)c1c(c23) ccc4c3ccc(=O)c4=O	255
133	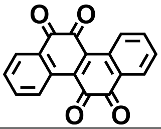	c1cccc(c(=O)c2=O)c1c(c23) c(=O)c(=O)c4c3cccc4	135
134	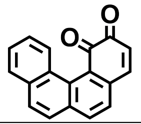	O=C1C(=O)C=Cc(c1c23)cc c3ccc4c2cccc4	1023
135	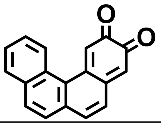	c1cccc(c1c23)ccc3ccc=4 c2=CC(=O)C(=O)C4	1023
136	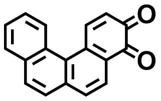	c1cccc(c1c23)ccc3ccc4 c2C=CC(=O)C4=O	1023
137	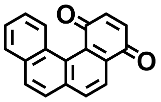	O=C1C=CC(=O)c(c1c23)cc c3ccc4c2cccc4	1023
138	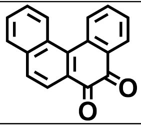	c1cccc(c1c23)C(=O)C(=O) c3ccc4c2cccc4	1023
139	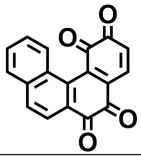	O=c1c(=O)ccc(c1c23)c(=O) c(=O)c2ccc4c3cccc4	255
140	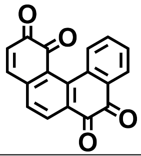	O=c1c(=O)ccc(c1c23)ccc3 c(=O)c(=O)c4c2cccc4	255

Continued on next page

Table S1 – Continued from previous page

No.	2D structures	SMILES	Number of functionalized derivatives
141	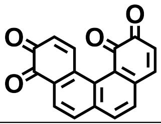	O=c1c(=O)ccc(c1c23)ccc3ccc4c2ccc(=O)c4=O	255
142	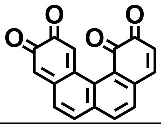	O=c1c(=O)ccc(c1c23)ccc3ccc4c2cc(=O)c(=O)c4	255
143	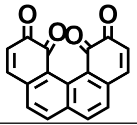	O=c1c(=O)ccc(c1c23)ccc3ccc4c2c(=O)c(=O)cc4	135
144	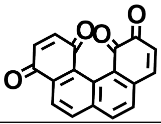	O=c1c(=O)ccc(c1c23)ccc3ccc4c2c(=O)ccc4=O	255
145	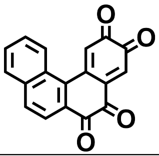	c1c(=O)c(=O)cc(c1c23)c(=O)c(=O)c2ccc4c3cccc4	255
146	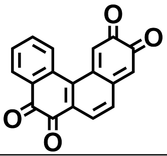	c1cccc(c1c23)c(=O)c(=O)c2ccc4c3cc(=O)c(=O)c4	255
147	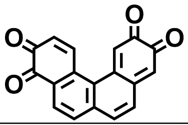	c1cc(=O)c(=O)c(c1c23)ccc3ccc4c2cc(=O)c(=O)c4	255
148	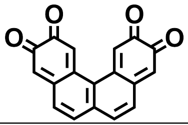	c1c(=O)c(=O)cc(c1c23)ccc3ccc4c2cc(=O)c(=O)c4	135
149	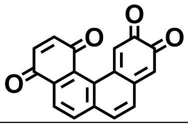	O=c1ccc(=O)c(c1c23)ccc3ccc4c2cc(=O)c(=O)c4	255
150	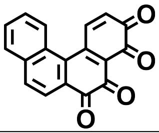	c1cc(=O)c(=O)c(c1c23)c(=O)c(=O)c2ccc4c3cccc4	255

Continued on next page

Table S1 – Continued from previous page

No.	2D structures	SMILES	Number of functionalized derivatives
151	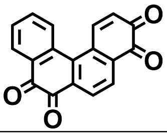	c1cc(=O)c(=O)c(c1c23)c cc3c(=O)c(=O)c4c2cccc4	255
152	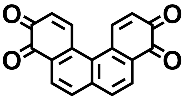	c1cc(=O)c(=O)c(c1c23)c cc3ccc4c2ccc(=O)c4=O	135
153	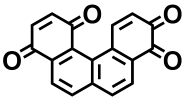	O=c1ccc(=O)c(c1c23)ccc3 ccc4c2ccc(=O)c4=O	255
154	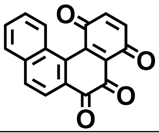	O=c1ccc(=O)c2c(=O)c(=O) c(c3c12)ccc4c3cccc4	255
155	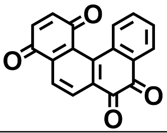	O=c1ccc(=O)c(c1c23)ccc3 c(=O)c(=O)c4c2cccc4	255
156	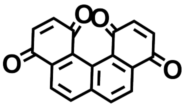	O=c1ccc(=O)c(c1c23)ccc3 ccc4c2c(=O)ccc4=O	135
157	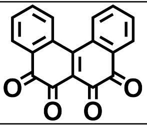	c1cccc2c1c(=O)c(=O)c(c23) c(=O)c(=O)c4c3cccc4	135
158	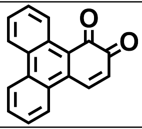	c1cccc(c1c23)c4c(cccc4) c2C=CC(=O)C3=O	1023
159	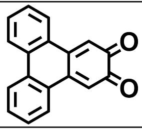	c1cccc(c1c23)c=4c(=C C(=O)C(=O)C4)c2cccc3	527
160	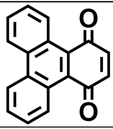	O=C1C=CC(=O)c(c12)c3 c(cccc3)c4c2cccc4	527

Continued on next page

Table S1 – Continued from previous page

No.	2D structures	SMILES	Number of functionalized derivatives
161	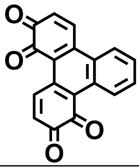	c1cc(=O)c(=O)c(c1c23)c4 c(cccc4)c2ccc(=O)c3=O	255
162	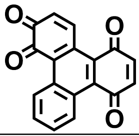	O=c1ccc(=O)c(c12)c3c (cccc3)c4c2ccc(=O)c4=O	255
163	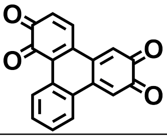	O=c1c(=O)ccc(c12)c3c (cc(=O)c(=O)c3)c4c2cccc4	255
164	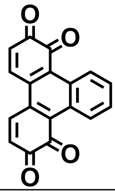	O=c1c(=O)ccc(c12)c3c(c(=O)c(=O)cc3)c4c2cccc4	135
165	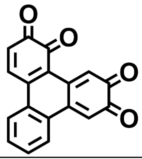	c1c(=O)c(=O)cc(c1c23)c4c (cccc4)c2ccc(=O)c3=O	255
166	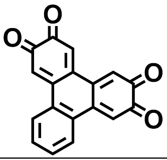	c1c(=O)c(=O)cc(c1c23)c4c (cccc4)c2cc(=O)c(=O)c3	135
167	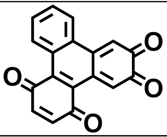	O=c1ccc(=O)c(c12)c3c (cccc3)c4c2cc(=O)c(=O)c4	255
168	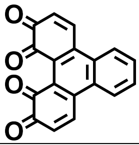	O=c1c(=O)ccc(c1c23)c4c (cccc4)c2ccc(=O)c3=O	135

Continued on next page

Table S1 – Continued from previous page

No.	2D structures	SMILES	Number of functionalized derivatives
169	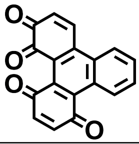	O=c1c(=O)ccc(c1c23)c4c (cccc4)c2c(=O)ccc3=O	255
170	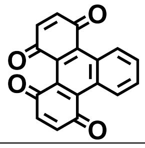	O=c1ccc(=O)c(c1c23)c4c (cccc4)c2c(=O)ccc3=O	135

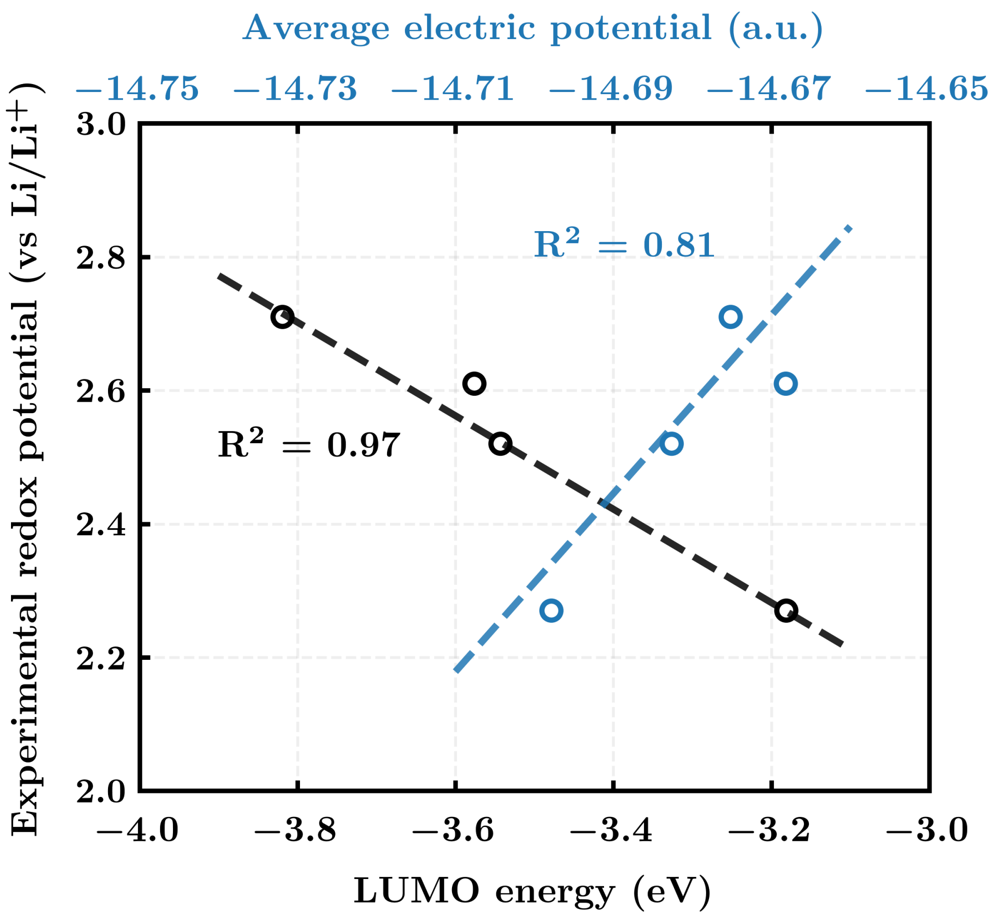

Figure S1: The prediction performance of LUMO energy of quinone molecule (black) and average electric potential of carbon atoms in carbonyl-equipped rings (blue).

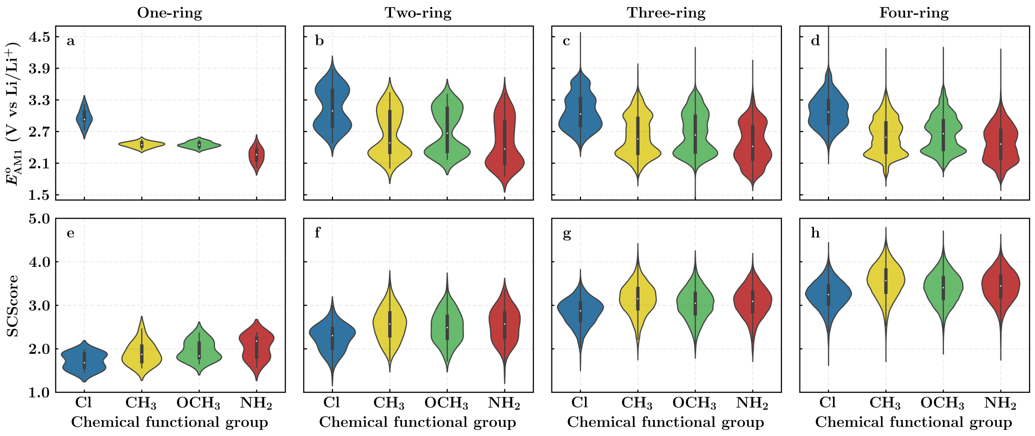

Figure S2: Violin plots showing distributions of E^{o}_{AM1} (a, b, c, d) and SCScore (e, f, g, h) data for approximately 200k candidates found in the virtual library. The distributions are shown for the type of chemical functional group that was present in (a, e) one-ring, (b, f) two-ring, (c, g) three-ring, and (d, h) four-ring molecules. The white dots that are in the center of the black bars represent the median values.

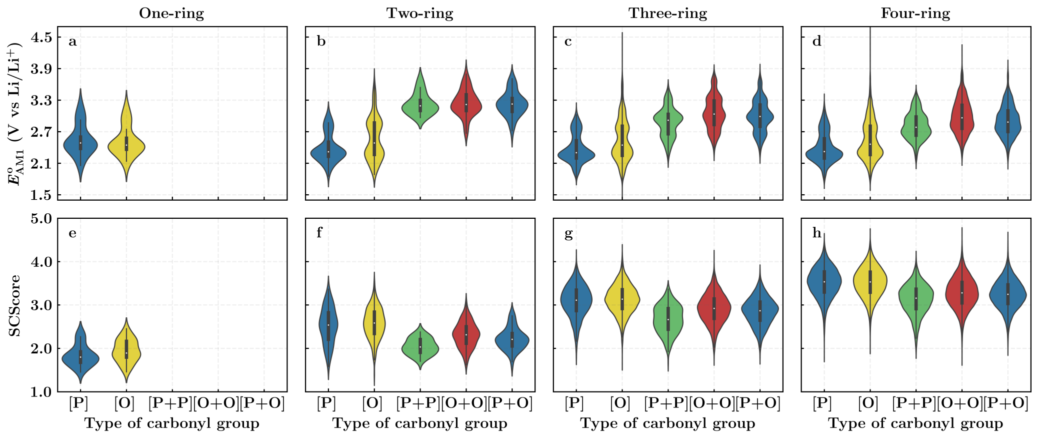

Figure S3: Violin plots showing distributions of E^{o}_{AM1} (a, b, c, d) and SCScore (e, f, g, h) data for approximately 200k candidates found in the virtual library. The distributions are shown for the type of carbonyl group that was present in (a, e) one-ring, (b, f) two-ring, (c, g) three-ring, and (d, h) four-ring molecules. The white dots that are in the center of the black bars represent the median values.

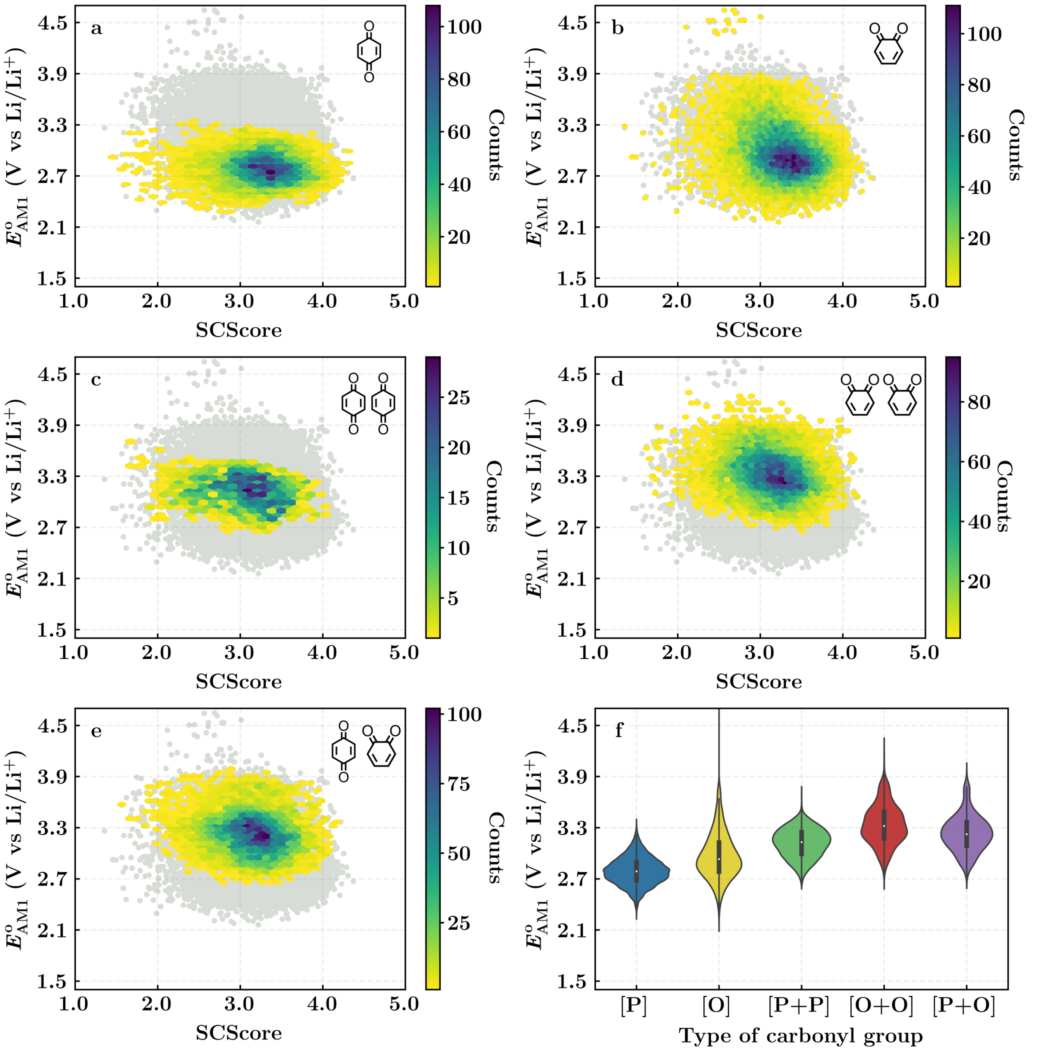

Figure S4: The distribution of the type of carbonyl group over the entire –Cl functionalized molecules, where the former are shown with colored dots and the latter with grey dots. The predicted E^{o}_{AM1} and SCScore values are shown for the compounds having (a) [P], (b) [O], (c) [P+P], (d) [O+O], and (e) [P+O] type of carbonyl groups in their CQSs. The color bars on the right show the number of molecules as indicated by the different colors. The violin plot (f) shows the distribution of the predicted E^{o}_{AM1} values with respect to the five different types of carbonyl groups found in the CQSs.

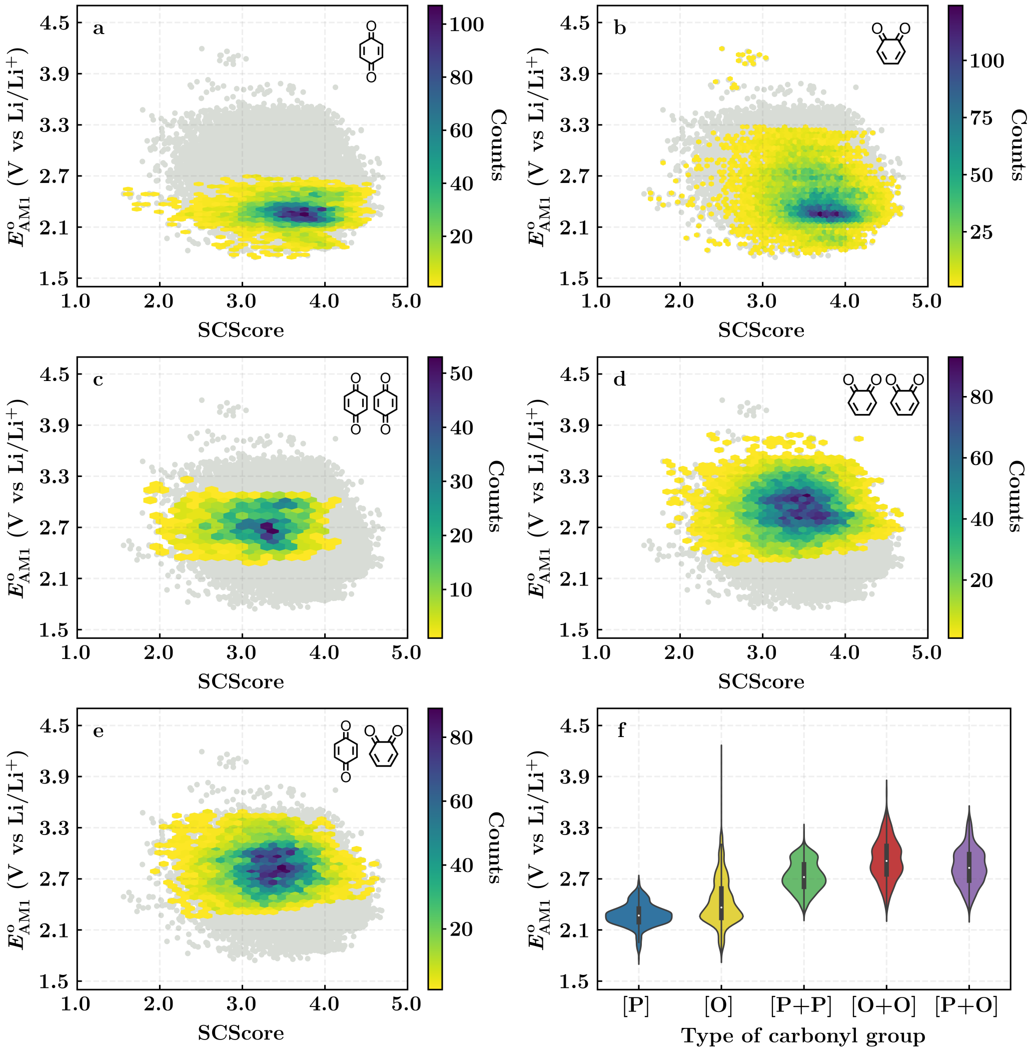

Figure S5: The distribution of the type of carbonyl group over the entire –CH_3 functionalized molecules, where the former are shown with colored dots and the latter with grey dots. The predicted E^{o}_{AM1} and SCScore values are shown for the compounds having (a) [P], (b) [O], (c) [P+P], (d) [O+O], and (e) [P+O] type of carbonyl groups in their CQSs. The color bars on the right show the number of molecules as indicated by the different colors. The violin plot (f) shows the distribution of the predicted E^{o}_{AM1} values with respect to the five different types of carbonyl groups found in the CQSs.

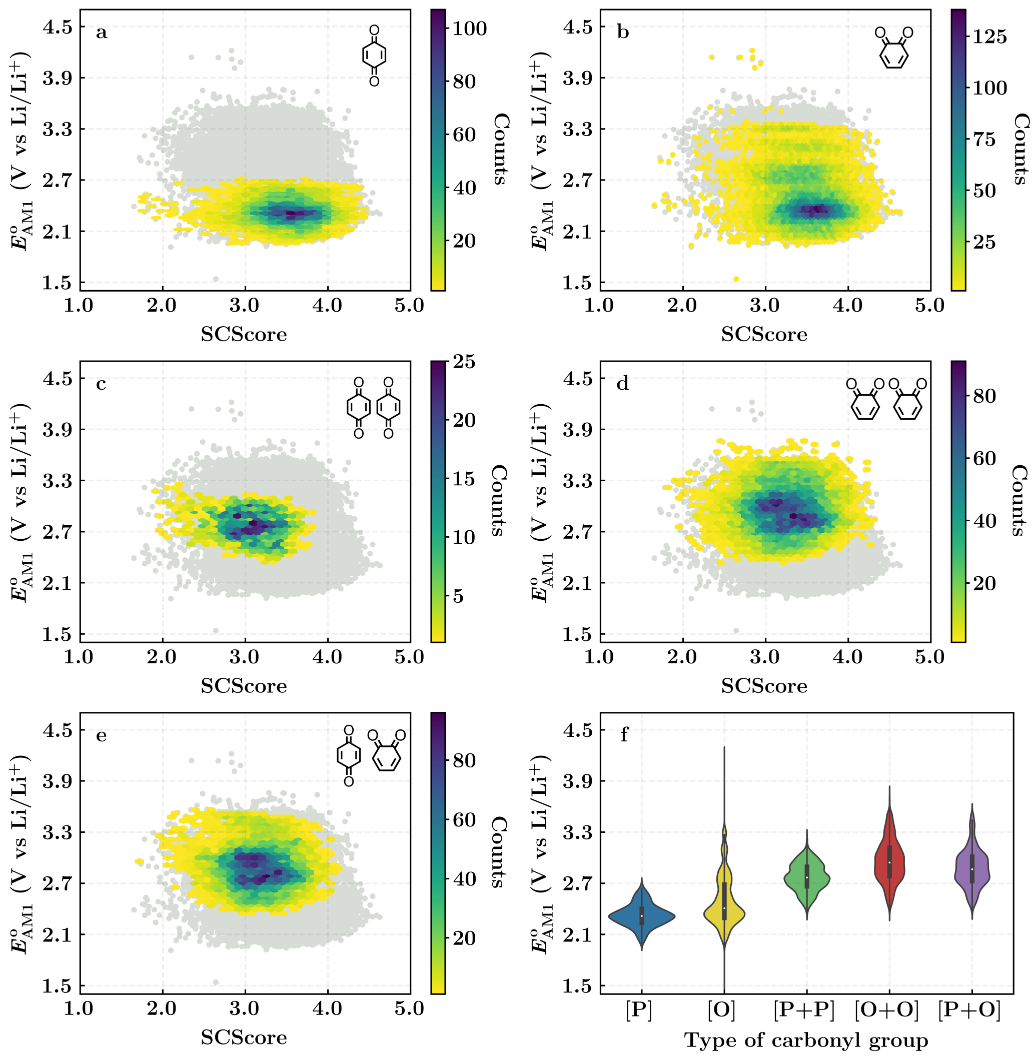

Figure S6: The distribution of the type of carbonyl group over the entire –OCH_3 functionalized molecules, where the former are shown with colored dots and the latter with grey dots. The predicted E^{o}_{AM1} and SCScore values are shown for the compounds having (a) [P], (b) [O], (c) [P+P], (d) [O+O], and (e) [P+O] type of carbonyl groups in their CQSs. The color bars on the right show the number of molecules as indicated by the different colors. The violin plot (f) shows the distribution of the predicted E^{o}_{AM1} values with respect to the five different types of carbonyl groups found in the CQSs.

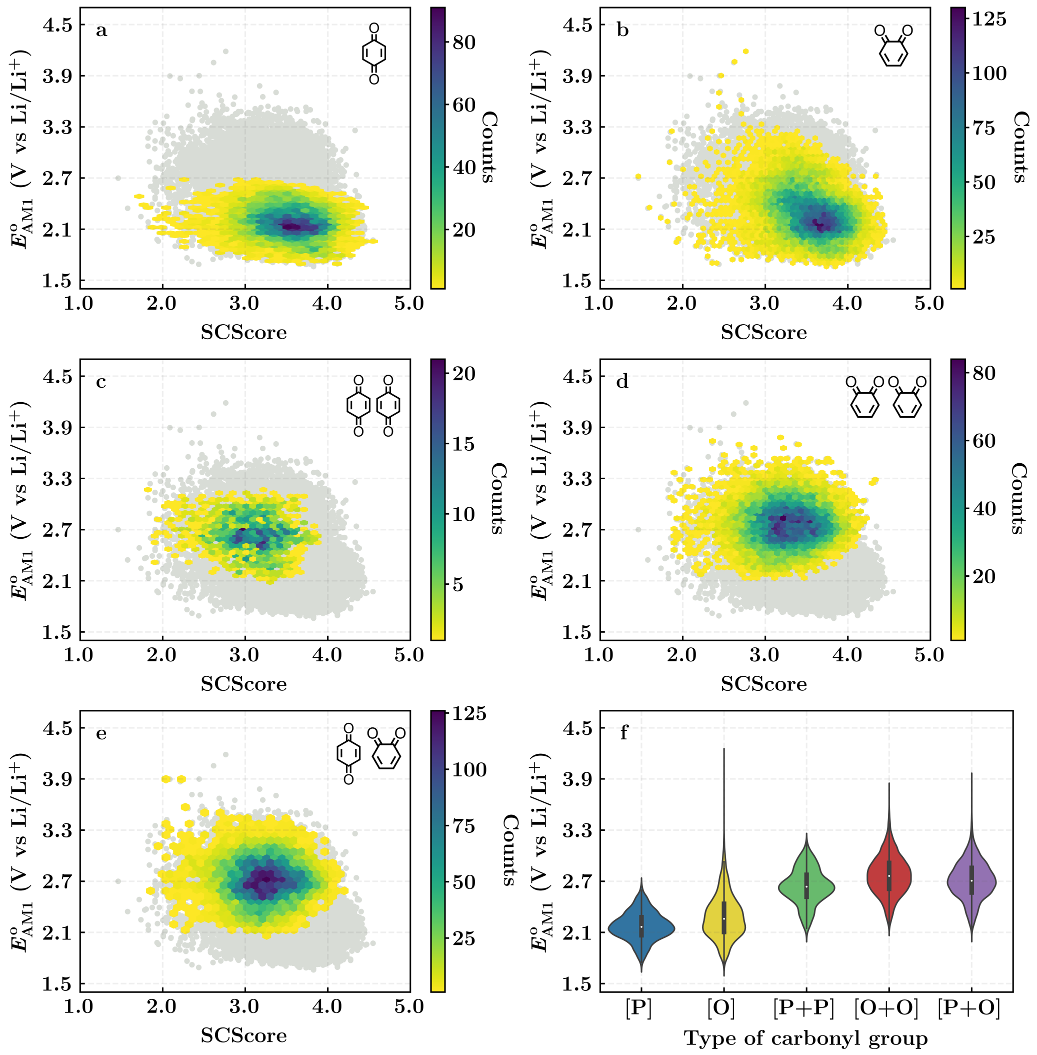

Figure S7: The distribution of the type of carbonyl group over the entire –NH_2 functionalized molecules, where the former are shown with colored dots and the latter with grey dots. The predicted $E^{\text{o}}_{\text{AM1}}$ and SCScore values are shown for the compounds having (a) [P], (b) [O], (c) [P+P], (d) [O+O], and (e) [P+O] type of carbonyl groups in their CQSs. The color bars on the right show the number of molecules as indicated by the different colors. The violin plot (f) shows the distribution of the predicted $E^{\text{o}}_{\text{AM1}}$ values with respect to the five different types of carbonyl groups found in the CQSs.
